# Unveiling hidden connections in omics data *via* pyPARAGON: an integrative hybrid approach for disease network construction

**DOI:** 10.1093/bib/bbae399

**Published:** 2024-08-20

**Authors:** Muslum Kaan Arici, Nurcan Tuncbag

**Affiliations:** Graduate School of Informatics, Middle East Technical University, Ankara 06800, Turkey; Chemical and Biological Engineering, College of Engineering, Koc University, Istanbul 34450, Turkey; School of Medicine, Koc University, Istanbul 34450, Turkey; Koc University Research Center for Translational Medicine (KUTTAM), Koc University, Istanbul 34450, Turkey

**Keywords:** network reconstruction, graphlets, data integration, interactome

## Abstract

Network inference or reconstruction algorithms play an integral role in successfully analyzing and identifying causal relationships between omics hits for detecting dysregulated and altered signaling components in various contexts, encompassing disease states and drug perturbations. However, accurate representation of signaling networks and identification of context-specific interactions within sparse omics datasets in complex interactomes pose significant challenges in integrative approaches. To address these challenges, we present pyPARAGON (PAgeRAnk-flux on Graphlet-guided network for multi-Omic data integratioN), a novel tool that combines network propagation with graphlets. pyPARAGON enhances accuracy and minimizes the inclusion of nonspecific interactions in signaling networks by utilizing network rather than relying on pairwise connections among proteins. Through comprehensive evaluations on benchmark signaling pathways, we demonstrate that pyPARAGON outperforms state-of-the-art approaches in node propagation and edge inference. Furthermore, pyPARAGON exhibits promising performance in discovering cancer driver networks. Notably, we demonstrate its utility in network-based stratification of patient tumors by integrating phosphoproteomic data from 105 breast cancer tumors with the interactome and demonstrating tumor-specific signaling pathways. Overall, pyPARAGON is a novel tool for analyzing and integrating multi-omic data in the context of signaling networks. pyPARAGON is available at https://github.com/netlab-ku/pyPARAGON.

## Introduction

Omics technologies provide a multidimensional view of the cell’s functional mechanism, context-specific alterations in diseases or drug perturbations, and biological processes [1, 2]. As the omics data accumulate, integrating them accurately and translating them into interpretable knowledge remains challenging due to data sparsity, missing data points, and computational complexity [3–5]. Omic hits are sparsely connected in a reference interactome and carry noise from high-throughput outcomes [6, 7].

Recent methods utilizing learning- and network-based algorithms are on the rise to overcome these challenges and decode causal relations between omic entities [8–11]. Learning-based methods efficiently integrate multi-omic data to extract interpretable annotations such as pathways, reactions, and processes [12–14]. Also, network-based algorithms, including shortest paths [15], Steiner trees/forests [16, 17], and random walks [18], have been frequently used to construct specific networks by propagating omic hits [19, 20]. Network-based methods can uncover the most relevant interactions between a given set of proteins/genes by either inferring from a reference protein–protein interaction (PPI) network or reconstructing them [1, 21, 22]. These reference networks integrate numerous databases and datasets, disregarding experimental context across diverse cell types and states [23]. Thus, the network inference methods may suffer from false positive interactions. However, these methods eventually obtain a network model, which may represent the alterations in disease models or the effects of drug treatments with the help of topological and statistical features [24–29]. The benefit of using global and local network features (e.g. degree distribution, clustering coefficients) for propagation or inference [30, 31] is limited when this type of sparse data is elaborated [32, 33]. Therefore, the frequent subgraphs, known as network motifs in biological networks such as metabolic [34], regulatory [35, 36], and cellular signaling networks [37], can provide a more comprehensive insight into their functional impact in complex cellular networks [38]. Specific network motifs function in rewiring signaling cascades and regulating cellular signaling and information processing, including feedback and feedforward loops, which entail signaling adaptations [39], cell lineage [40], and cell dynamics and functions in tissue [41]. Small connected, non-isomorphic subgraphs, called *graphlets*, are over-represented in the reference interactome and associated with specific functions [42, 43]. Graphlet statistics solve several complex problems in this context, such as the comparison of biological networks, delineating the functional organization of networks, discovering functionally related genes, regulatory interactions, and parameter tuning for network-based approaches [12, 32, 33, 44–47]. Another challenge is the presence of highly connected and multifunctional proteins, particularly hub proteins, which can bring nonspecific interactions to the resulting network models. Therefore, using network motifs, graphlets, or revealing modules can improve the context-specific aspects of the models [1, 25, 48].

In this study, we hypothesize that the utilization of network motifs, *in lieu* of pairwise connections among proteins, may provide a more accurate representation of signaling networks and mitigate the inclusion of nonspecific interactions. Therefore, we present pyPARAGON (PAgeRAnk-flux on Graphlet-guided network for multi-Omic data integratioN) that combines network propagation with graphlets to construct context-specific networks. We found that graphlets filter out nonspecific interactions and mitigate the dominance of highly connected nodes, thereby trimming the reference interactome. pyPARAGON, as a hybrid method, performed better than the selected state-of-the-art methods in the reconstruction of known cancer signaling pathways. We demonstrated the utility of pyPARAGON in patient stratification using a breast cancer dataset comprising 105 tumors and associated phosphoproteomic data. Our analysis unveiled tumor-specific signaling pathways for each patient group.

## Methods 

### Overview of pyPARAGON as a hybrid network inference framework

**Figure 1:**
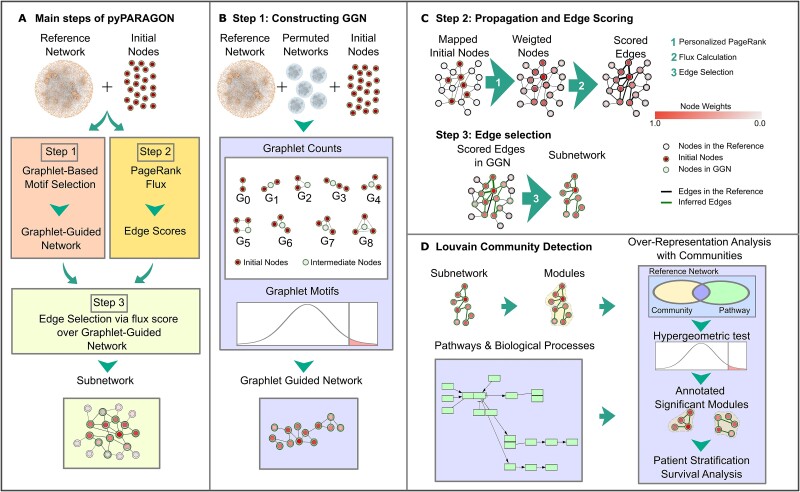
The overview of pyPARAGON. (A) pyPARAGON uses a reference network and a set of initial nodes (seed nodes) as the input. pyPARAGON has three steps: (i) GGN construction; (ii) edge scoring with PPR flux calculation; (iii) subnetwork inference using edge scores and GGN. (B) We investigated nine non-isomorphic graphlets (G_0_–G_8_) composed of 2, 3, and 4 nodes in the reference network and its 100 permuted networks. Except for G_0_, each graphlet covers at least two seed nodes (red circles) and one intermediate node (white circles) that connects the seeds in the center of the orbit. We conducted a z-test to compare the frequency of graphlets in the reference and permuted networks. The union of significantly frequent graphlets constructs GGN. (C) By random walking from weighted initial nodes in the reference network, the PPR algorithm assigns weight to each node during propagation. Then, computed edge fluxes were used as the edge scores in the reference interactome. In the edge selection step, high-scoring ones in GGN construct the final subnetwork. (D) pyPARAGON employs the Louvain community detection method, based on network topology, to divide the inferred network into functional units. Significant biological processes and pathways in each module were found by using a hypergeometric test.

Hybrid approaches can be more effective than relying on a single method alone when integrating different types of omic data [17]. The accuracy of reconstructed networks is highly dependent on the reference interactome quality [49, 50]. On one hand, including interactions with low confidence scores may lead to the identification of false positive proteins and interactions. On the other hand, highly connected proteins (i.e. hubs) may dominate the final network and obscure context-specific connections of proteins/genes. pyPARAGON copes with these challenges in two independent steps. First, graphlet search mitigates the dominance of hub nodes. Graphlets are small, connected subgraphs with a specific pattern of edges and are similar to network motifs representing recurring patterns [42, 43]. Additionally, pyPARAGON calculates the flux value by multiplying a node’s propagation score with the confidence score of its interaction and normalizing it with its degree. In this way, pyPARAGON prioritizes the high confidence scores and associated nodes while penalizing the highly connected nodes*.* pyPARAGON has three steps (Fig. 1A): (i) graphlet-guided network (GGN) construction; (ii) propagation and edge scoring *via* the Personalized PageRank (PPR) algorithm and flux calculation; (iii) preserving the edges in GGN with high scores and filtering out the rest.

In general, state-of-the-art methods use an immediate edge between two nodes in the reference network and node-based features (e.g. degree, betweenness, closeness, and eigenvector centralities). The GGN construction step of pyPARAGON goes further by following an unsupervised approach to identify a core region in the reference interactome by combining significantly frequent graphlets composed of 2-, 3-, and 4-nodes (Fig. 1B). In omics-based network construction, direct connections between the genes/proteins of interest are often sparse, and intermediate nodes are required to connect them and form a coherent network structure. Thus, we constrained that graphlets having more than two nodes may have an intermediate node. Intermediate nodes are the ones that have the highest connections to the seed nodes (initial nodes) in the corresponding graphlet (Fig. S1A).

In the second step, the PPR algorithm propagates signals from seed nodes across the reference interactome. Node weights after propagation, their degrees, and edge confidence scores are combined in a single function to calculate edge fluxes [51]. If the reference interactome is an unweighted graph, pyPARAGON sets a default score of 1.0 for all edges. Similarly, if seed nodes do not have weights, pyPARAGON assigns them a default value of 1.0. In this function, the degree component penalizes highly connected proteins that are nonspecifically present in the resulting subnetworks. In the final step, we map edges with flux scores to GGN to obtain a context-specific network (Fig. 1C). To simplify biological interpretation, pyPARAGON additionally uncovers modules, corresponding to network communities, which function in specific biological processes or pathways (Fig. 1D). Based on network topology, the Louvain community detection method divides inferred subnetworks into small modules [52]. Then, using a hypergeometric test, pyPARAGON discovers context-specific annotations [53]. In this way, we reveal not only hidden connections between initial nodes but also significant context-specific modules.

### Network inference via PageRank-flux on graphlet-guided network

We used 2-, 3-, and 4-node-graphlets (*G_0_, G_1_, G_2_, …, G_8_,* shown in Fig. S1A), which are small non-isomorphic subgraphs. An isomorphism of graphlets between two subgraphs, *X(V_X_, E_X_)* and *Y(V_Y_, E_Y_)*, is defined with bijections between *V_X_* and *V_Y_* [42]. We searched the graphlets for an intermediate node in one of the highest-degree orbits and seed nodes in the remaining orbits. The reference network is *R*(*V_R_, E_R_, c(e)*), where *V_R_*, *E_R_,* and *c(e)* are node set, undirected edge set, and their confidence scores, respectively. Similarly, we calculated the frequencies of graphlets in 100 permuted networks, recruiting the same seed node set. To prepare permutated networks, we randomly swapped two edges between four different nodes so that the network topology and the number of interactions of the reference interactome could be used for statistical analysis [21]. We compared the graphlet frequencies in the reference and permuted networks with a z-test (*P* < 0.05, z-score > 1.65). The union of significant graphlets constructs the GGN, *G(V_G_, E_G_),* where *G ⊆ R*.

The PPR algorithm calculates the probability of being at each node at a particular step in the reference networks according to Equation (1).


(1) \begin{align*} {p}_{\left(t+1\right)}(y)=\frac{1-\lambda }{N}+\lambda \sum_{y={x}_i}^{x_N}\frac{p_t\left({x}_i\right)}{{deg}\ \left({x}_i\right)\ } \end{align*}

where *p(y)* represents the probability of being at node *y* in the network at a particular time step *t* and *λ* is the damping factor. *x_i_* represents each neighbor of *y*. *deg(x_i_)* is the degree of node *x_i_* and *N* is the number of nodes [54, 55].

We modified the combined score formula described by Rubel and Ritz and introduced directed flux scores accordingly [51]. When combining two directional flux scores, we assigned the minimum flux score to the edge, instead of multiplying both directional flux scores. The scores are calculated for both directions (*f_u → t_* and *f_t → u_*) by using Equations (2 and 3), where *u, t ∈ V_R_*, and *e* is the edge between *u* and *t*, respectively. The negative logarithm of minimum flux scores is used as a final edge score (*f(e)*) defined in Equation (4).


(2) \begin{align*} {f}_{u\to t}\left(u,t\right)=\frac{p(u).c(e)}{{deg}(u)} \end{align*}

(3) \begin{align*} {f}_{t\to u}\left(t,u\right)=\frac{p(t).c(e)}{{deg}(t)} \end{align*}

(4) \begin{align*} f(e)=-{log}\ \left({min}\left({f}_{u\to t}\left(u,t\right),{f}_{t\to u}\left(t,u\right)\right)\right) \end{align*}

We weighted the edge set of GGN, *G(V_G_, E_G_)*, with *f(e)* where *e_1_, e_2_, e_3_, …, e_j_, …e_n_ ∈ E_G_*, *1 ≤ j ≤ n* and *f(e_j-1_) > f(e_j_) > f(e_j + 1_).* The total flux scores (*F*) in GGN are calculated as formulated in Equation (5).


(5) \begin{align*} F=\sum_{i=1}^nf\left({e}_i\right)\ (5) \end{align*}

Let *τ* (*0 ≤ τ ≤ 1*) represent the scaling factor describing the threshold percentage of *F*. We selected the edges by summing flux scores up to *τxF* (Equation (6)). In this way, we infer the context-specific network *C(V_C_, E_C_),* where *E_C_ ⊆ E_G_* and *V_C_ ⊆ V_G_*.


(6) \begin{align*} \tau xF=\sum_{i=1}^jf\left({e}_i\right),1\le j\le n\ \end{align*}

## Results

### Network trimming via graphlets improves network inference

We used NetPath [56] as the benchmark dataset to reconstruct curated signaling pathways and assess the performance of pyPARAGON. In general, the performance of the methods is evaluated based on topological features, coverage of predicted nodes, and edges. As a result of screening all graphlets across the reference interactomes, we found *G_2_*, *G_5_*, *G_6_*, *G_7_*, and *G_8_* to be the most frequent graphlets (Fig. S1A). The frequency of direct interactions between input nodes (represented with *G_0_*) is insignificant in the reference interactome; however, the direct interactions in a graphlet with at least three nodes are significant. For example, the direct interaction of seed nodes in *G_2_* gets more important in the presence of an intermediate node interacting with *G_0_*. As to our observation, significant graphlets having at least one intermediate node to connect seeds provide more precision compared to including direct interactions between two seeds (i.e. *G_0_*) in GGN.

**Figure 2:**
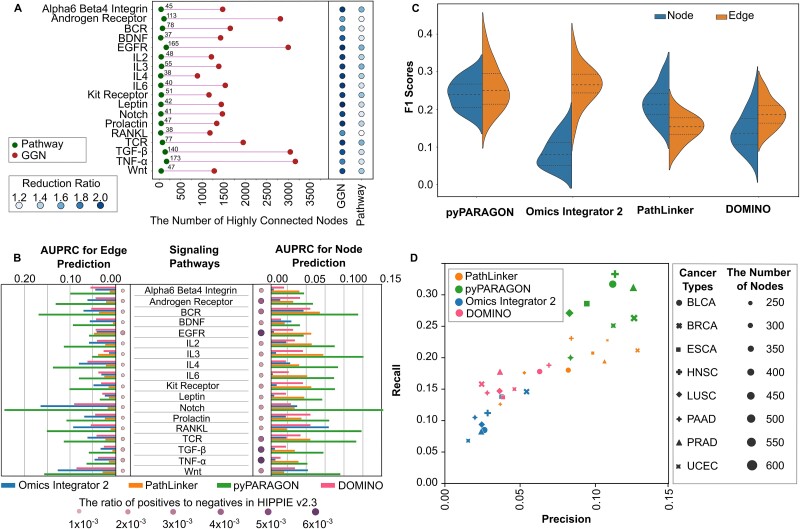
GGN trims reference interactome by removing some highly connected nodes and their non-specific interactions. (A) Highly connected nodes (3887) are defined with degrees within the top 20% of all proteins in HIPPIE interactome. On the left side, the presence of these nodes in GNNs and reconstructed pathways is shown for each signaling pathway (red and green dots, respectively). On the right side, the reduction ratio (RR) separately represents the decrease in the interaction number of highly connected nodes for GGN and pathways. (B) AUPRC of each tool (blue = OI2, orange = PL, red = DOMINO, and green = pyPARAGON; left panel for edge prediction and right panel for node prediction performance) in each pathway reconstruction. The ratio of positives to negatives in HIPPIE interactome is scaled at 10^−3^, which demonstrates the sparsity of target nodes and edges in the reference network. (C) Distribution of F1-scores for each tool across 18 pathways is shown for node (blue) and edge (orange) predictions. (D) Performance evaluation in cancer-specific networks for eight distinct cancer types. Marker size represents network sizes, while recall and precision scores are shown on the x-axis and y-axis. The recall score represents the ratio of correctly predicted cancer driver genes in cancer-specific networks to the total number of drivers.

Each available interactome has a specific evaluation and scoring scheme to integrate PPIs from different resources [49]. In this study, we used ConsensusPathDB [57], HIPPIE v2.2, and HIPPIE v2.3 [58], which have different topological features (Supplementary Methods*,*  Table S1). The constructed GGN by pyPARAGON is a subnetwork of the reference interactome. When we separately compared the original interactomes and trimmed interactomes via GGN construction, we observed that their similarities significantly increase when GGNs are used (Fig. S1B). Another advantage of GGN construction is attenuating the dominance of the highly connected nodes with degrees within the top 20% of all nodes in the reference network [59]. Notably, highly connected proteins have numerous functions in the cellular processes known from prior knowledge and interactions in reference networks [60]. pyPARAGON puts a constraint on graphlets in that seed nodes must be connected via an intermediate node. The constructed GGN eventually consists of the topologically most important part of the reference interactome. Thus, nodes that are not present in any graphlet and not linked to the seed nodes, are trimmed. With this approach, we eliminate the most highly connected nodes (3887) in the reference network, HIPPIE v2.3 (Fig. 2A). The remaining highly connected nodes within GGN lost a large number of interactions that are not related to the given context (Fig. S1C). Also, the final GGNs preserve the properties of a scale-free network (Fig. S1D) [57], which characterize biological networks where the distribution of node degrees follows a power-law distribution [61, 62]. However, based on their degree exponent (γ_HIPPIE_ = 1.45 R^2^_HIPPIE_ = 0.88, and γ_GGNs_ = 2.40, R^2^_GGNs_ = 0.84), GGNs have stronger features of a scale-free network [23, 63, 64]. Scale-free networks are robust to the random loss of nodes, defined as error tolerance, and fragile to targeted worst-case attacks [65].

We compared the performance of pyPARAGON with three selected state-of-the-art tools, PathLinker [15], Omics Integrator 2 [16], and DOMINO [17]. PL computes multiple shortest paths between seed nodes and selects the highly-scored interactions by maximizing their own path scores to get rid of the noise. Omics Integrator 2 (OI2) and DOMINO solve the prize-collecting Steiner Forest problem. DOMINO statically selects the most relevant interactions and then solves the prize-collecting Steiner tree problem. These four approaches are compared based on their performance in inferring curated signaling pathways in NetPath. Since there is no definitive benchmark or ground truth for assessing tool performance, we relied on propagated nodes and predicted edges as evaluation criteria. Since the performance of tools is highly dependent on parameter sets, we inferred signaling pathways by applying various parameter sets in a grid for each network inference tool (Supplementary Methods). Then, we measured performance using the area under the precision-recall curve (AUPRC) to demonstrate how well each pathway’s nodes and edges were recovered in the predicted networks. Bias toward hub proteins in the reference interactomes is a challenge in signaling pathway reconstruction that has been considered in all (pyPARAGON, OI2, PL, and DOMINO). Our analysis showed that pyPARAGON outperformed these tools at both the node and edge levels for inferring signaling pathways in all pathways of NetPath (Fig. 2B). Furthermore, the proportion of positive and negative instances, based on both nodes and edges, indicated that our target nodes and edges are extremely scarce inside the reference network (Table S2).

Performance comparison of pyPARAGON with others was done in two directions: (i) node propagation, (ii) edge inference. We used the F1 score to compare them because it simultaneously represents precision and recall in one metric. The overall results show that pyPARAGON and PL are better at propagation, while pyPARAGON and OI2 are better at network inference (Fig. 2C). Due to the usage of significant modules in reference networks, DOMINO runs in a balance to propagate nodes and predict interactions. These modules are defined based on annotations in Gene Ontology. However, missing annotations in reference networks and databases may lead to low-performance scores. Thus, DOMINO exhibits lower F1 scores and AUPRC than pyPARAGON. Highly connected reference networks decreased the propagation ability of OI2 while providing more robust interactions than PL. On the other hand, PL propagated the seed node set more robustly due to considering multiple shortest paths but introducing many false positive interactions. Many seed nodes have a tendency to be connected by hub nodes as shortcuts due to biological networks being scale-free. Thus, multiple-shortest paths and random walk-based approaches may include more false positive interactions [19, 66]. However, penalizing highly connected nodes, e.g. the calculation of PageRank flux normalized the score in pyPARAGON or degree-dependent negative prizing in OI2, reduces false positive edges and improves F1-score in edge prediction.

Cancer driver genes provide a selective growth benefit and enhance cancer development via harboring specific mutations. Therefore, predicting and prioritizing genes likely to play a crucial role in oncogenesis are important tasks. We next utilized pyPARAGON to construct cancer network models to test its performance in detecting driver genes. The most frequently mutated genes in eight cancer types are utilized as seed nodes [67, 68]. We compared the nodes in the reconstructed networks with the known driver genes in IntOGen database [69] (Supplementary Methods), listed in pyPARAGON/Supplementaries folder on GitHub. Because we use 5-fold cross-validation, for each fold, we filtered out the common proteins between the seed list and known drivers and then reconstructed cancer type-specific networks with pyPARAGON, PL, DOMINO, and OI2.

Cancer-type-specific networks include both driver gene nodes and the intermediate nodes. However, not all cancer driver mutations, genes, and functionalities are known in the available datasets. Consequently, the accuracy of predicting driver genes in the absence of ground truth is the reason of low performance metrics, particularly in precision scores. As shown in Fig. 2D, the reconstructed network by pyPARAGON finds more driver genes and mostly achieves higher recall and precision than other methods in all cancer types (Supplementary Table S3). In PL-generated networks, precision scores are in general close to pyPARAGON. They are better than pyPARAGON for ESCA and BRCA. PL recruits the multiple shortest paths. Thus, intermediate nodes corresponded more to highly connected genes than specific driver genes with default parameters. In pyPARAGON, we use the PageRank algorithm to propagate seed nodes to the neighbors in the reference interactome, which helps obtain more candidate drivers. The prize-collecting Steiner tree algorithm terminates propagation at the seed nodes, which results in fewer driver genes being recovered in networks inferred by OI2 and DOMINO. In large reference networks, highly connected nodes generate network shortcuts instead of using signal cascades or motifs. Overall, pyPARAGON performs significantly better in cancer driver network prediction and can be further elaborated for tumor- or patient-specific network construction and network similarity-based comparisons.

### Tumor-specific network inference unveil hidden commonalities across patients

**Figure 3:**
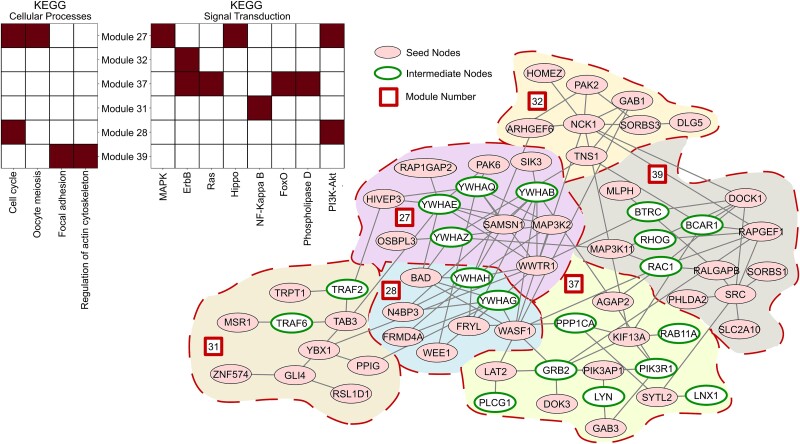
An example active module in tumor-specific network constructed by pyPARAGON (TCGA-A8-A079). Significantly phosphorylated proteins were used as the initial (seed) node-set (colored pink), and intermediate nodes predicted by pyPARAGON are in green circles. Active modules, bordered with dashed red lines and numbered within red boxes, are associated with at least one significantly overrepresented KEGG pathway. The pathways belonging to cellular processes and signal transduction are shown in the top left panel.

**Figure 4:**
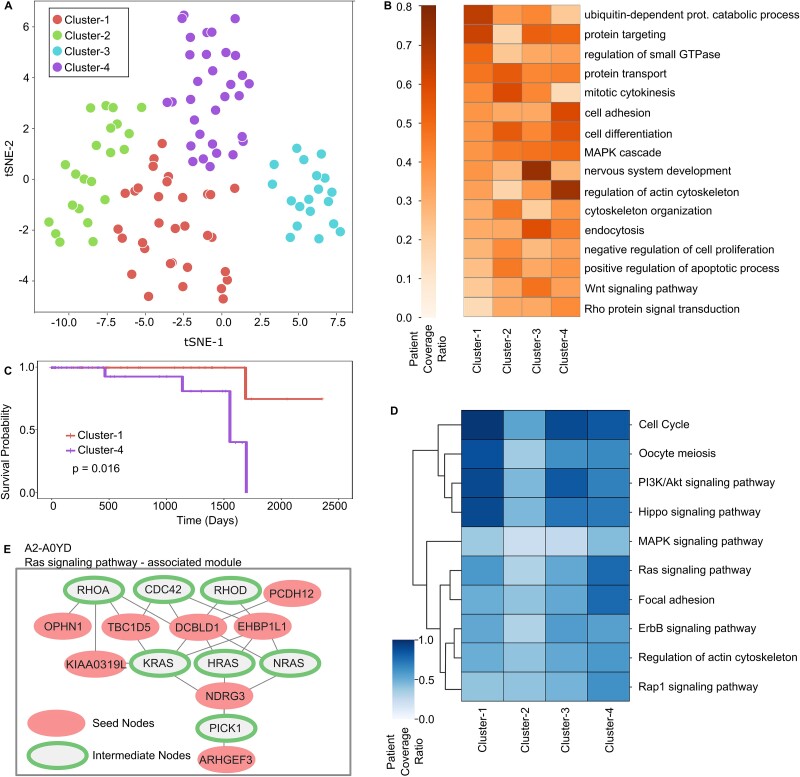
Stratification of tumors and associated biological processes with patient clusters. (A) 105 breast cancer tumors are stratified into four clusters based on their similarity of significant biological processes in their network modules: Cluster-1 (32 patients), cluster-2 (22 patients), cluster-3 (19 patients), and cluster-4 (32 patients). (B) Heatmap of patient coverage ratio for each cluster and significant process pairs. A biological process is included in the heatmap if it is enriched in at least two clusters. The patient coverage ratio represents the ratio of patients having the enriched biological process in the corresponding clusters. (C) Kaplan–Meier analysis shows the survival probabilities of cluster-1 (red) and cluster-4 (purple). The log-rank test (*P* < 0.05) statistically demonstrated that patients in cluster-4 have a significantly worse survival probability than cluster-1. (D) Heatmap shows significantly enriched KEGG pathways in active modules. (E) The example module of the A2-A0YD-specific network corresponding to the Ras signaling pathway is shown where seed nodes are red and intermediate nodes are green.

We employed pyPARAGON to construct the specific networks for 105 breast cancer patients’ tumors [68], where the seed nodes are significant phosphoproteins, as detailed in Supplementary Methods. It is important to note that pyPARAGON is also applicable to pan-cancer datasets. We consider the modules as functional subunits of networks that participate individually or jointly in context-specific molecular processes (Supplementary Methods). pyPARAGON uses hypergeometric tests to identify these active modules that are significantly over-represented in specific biological processes (Fig. S2). Figure 3 shows an example tumor-specific network composed of active modules that are significantly associated with KEGG pathways. All modules of the tumor-specific network are visualized and demonstrated in Fig. S3. Similarly, we identified active modules annotated with biological processes and then calculated the cosine similarities between patient-specific networks. Eventually, patient tumors are clustered into four groups (Fig. 4A, Supplementary Methods). Table S4 lists the 20 most common biological functions for each cluster. We uncovered critical biological processes in at least two clusters (Fig. 4B). In patient cluster-1, the most frequently associated biological process is the ubiquitin-dependent protein catabolic process, where several transcription factors (TFs) and enzymes are present. Ubiquitination (one of the post-translational modifications) is a multistep enzymatic process involved in the regulation of cancer metabolism [70]. The patients in cluster-2 frequently share the mitotic cytokinesis process. Cytokinesis defects increase chromosomal instability, vast genomic alteration, and point mutations, provoking intratumoral heterogeneity [71, 72]. The patient similarity network (Fig. S4) shows that only five patients in cluster-2 have higher similarity scores than 0.5 due to heterogeneity. Interestingly, we found that the nervous system development (NSD) process was the most frequent biological process in cluster-3. Breast cancer is the second most common cause of central nervous system metastasis after lung cancer [73]. In our datasets, just two patients had metastases. We found both patients with the NSD process in cluster-3. In cluster-4, the regulation process of actin cytoskeleton organization is significantly enriched which is relevant to cancer initiation, metastasis, and therapeutic responses. Rho GTPases, a family of the Ras GTPase superfamily, play a key role in this regulation [74].

Survival and KEGG pathway over-representation analysis revealed distinct molecular variations among clusters through tumor-specific signaling pathways. The Kaplan–Meier analysis and the log-rank test of the overall survival of patient clusters [75] showed that patients in cluster-4 have a significantly worse survival probability than cluster-1 (Fig. 4C, Fig. S5). Followingly, we annotated active modules with KEGG pathways to figure out their over-representation in these clusters (Fig. 4D) [76]. Cell cycle and PI3K/Akt signaling pathways are the most frequent pathways in the clusters, except cluster-2. Their presence in tumors in cluster-1 is more frequent than in cluster-4. Critical protein complexes in DNA replication, repair mechanisms, and mitosis; Cyclin-dependent kinases (CDKs) regulate the cell cycle pathway [77]. Dysregulation of CDKs in breast cancer mediates changes in cell cycle progression, driving uncontrolled cell proliferation [78]. Additionally, CDKs mediate crosstalk between PI3K/Akt and cell cycle pathways [79]. Thus, CDKs and their regulators have become prominent targets for drug development [80]. We identified 23 TFs from TRRUST database [81] regulating CDKs in tumor-specific networks (Table S5). Ninety drugs, authorized by the FDA in the Therapeutic Target Database (TTD) [82], target eight of these TFs (Table S6). On the other hand, the activation and inactivation in the components of the Hippo signaling pathway lead to drug resistance through rewiring in cell cycle cascades [83, 84]. The focal adhesion and Ras signaling pathways are significantly more frequent in cluster-4. The Ras signaling pathway is one of the key pathways for drug resistance owing to the bypassing of drug action mechanisms in the signaling network [85, 86]. In Fig. 4E, we demonstrated the module associated with the Ras-signaling pathway, where pyPARAGON linked phosphoproteins with intermediate nodes, including KRAS, NRAS, HRAS, RHOA, and RHOD. Next, we extracted 8297 drugs, 330 drug targets from TTD [82], and active modules were linked to 161 pathways that are found significantly enriched in 105 breast cancer patient-specific networks (on GitHub at pyPARAGON/Supplementaries/). An example of drugs connected to the active modules of patient A2-A0YD is given in Fig. S6. Adagrasib (MRTX849) and Sotorasib specifically target the Ras signaling-associated module. Both drugs are novel KRAS^G12C^ inhibitors approved by the FDA [85, 87].

## Discussion

In this work, we present pyPARAGON as a network-based multi-omic data integration tool. pyPARAGON simultaneously utilizes the most frequent graphlets covering omic hits and network propagation to construct context-specific networks. Network inference algorithms encounter challenges arising from sparse data and the complexity associated with the growing number of interactions within reference networks and potential false positives in the inferred subnetwork. In our study, by employing pyPARAGON, we mitigated the impact of highly connected nodes in the reference networks. pyPARAGON eliminates the interactions based on the calculated edge fluxes. Thus, the reference interactome is not prefiltered based on a confidence threshold. Additionally, pyPARAGON preserves scale-free properties inherent in biological networks in the constructed GGNs. We leveraged the PageRank flux calculation for edge prioritization and integrated GGNs to successfully construct context-specific networks. Additionally, driver networks that are inferred by pyPARAGON encompassed more precise and higher number of cancer drivers.

Although network inference algorithms can infer context-specific networks from large reference databases and experimental data, these networks prevent complete biological interpretations. Thus, module identification is crucial for gaining biological interpretations from network knowledge. Independent of the network inference, pyPARAGON is able to identify functional modules and their corresponding annotations. In tumor-specific networks, we integrated modules and different types of annotations, such as biological processes in GOA, pathways in KEGG, and drug knowledge in TTP. Additionally, we statistically explored interpretable biological knowledge in modules to disentangle tumor-specific pathways. These annotations can be valuable in revealing commonalities and differences across patients and drug perturbations.

Different molecular aberrations representing the context can induce identical disease outcomes [88–90]. pyPARAGON was developed as a general-purpose framework for integrating a given list of proteins/genes or other biological entities from any omic resources and an interactome for various contexts. In this study, we used omics data from CPTAC breast cancer samples [68] to infer context-specific networks with annotated modules. Patients were clustered based on the network-based similarity between the overrepresented biological processes identified with functional modules. We show that active modules with the same driver genes mediate various biological processes or pathways. Thus, pyPARAGON potentially enhances the identification of hidden functional commonalities beyond the common edges and nodes across context-specific networks.

pyPARAGON provides advantages for multi-omics data integration strategies as well. For instance, significantly expressed genes that are identified from transcriptomic data, can be used for identifying significantly active TFs and providing as a part of input seed sets. For the same example, the list of mutated proteins and significant TFs can be used together to form a seed node list. Similarly, enzymes or substrates associated with metabolomic hits can be extracted to be used as inputs, if available. Proteomics or phosphoproteomics hits can be used directly as the seed node set. In another independent case study, we determined differential TFs between cancer and autism spectrum disorders by identifying their common target pathways, using transcriptomics hits and frequent mutations [91]. Differential TFs in disease-specific networks demonstrated how rewired signaling mechanisms alter disease phenotypes [91]. All these case studies proved that pyPARAGON is capable of integrating omic datasets via networks. pyPARAGON can be used to integrate various datasets, including the data from Pan-Cancer Atlas [92], the Cancer Cell Line Encyclopedia (CCLE) [93], the Genomics of Drugs Sensitivity in Cancer (GDSC) [94], and the LINCS [95], to reveal new biological insights in complex diseases, and drug perturbations.

Recent network inference methods, such as the SWEET tool [23], aim to construct sample-specific networks for individual samples [96–98]. pyPARAGON is highly modular, and the users can provide a custom reference interactome as input. For example, an aggregated interactome generated by SWEET to cover all phenotypic alterations across the entire sample set can be given as the reference. Then, pyPARAGON can infer a final subnetwork for each sample.

Despite the success of integrative approaches, including pyPARAGON, there is still significant potential for further enhancement.. Notably, network-based methods strongly depend on the features and coverage of reference networks [99]. As a result of incomplete knowledge in large reference interactomes, protein complexes tend to form more topological modules than metabolic pathways [100, 101]. Thus, generic biological processes, such as transcription and replication, can be found more frequently in inferred networks. Thus, biological interpretations of context-specific networks are challenging through causal relations, modules, and biological processes. Additionally, some network-based methods cannot handle the alternative copies of individual hits e.g. various protein isoforms and different post-translational modifications of a protein. Despite delivering more specific functions, this information is generalized and potentially lost in the network.

Extended integrations in reference networks and highly connected nodes have become a prominent challenge in recent network inference tools based on belief propagation [102, 103], random walks [18, 104], the prize-collecting Steiner Forest [16, 105, 106], heat diffusion [107, 108], and shortest path algorithms [15, 109]. Here, graphlets were deployed in our approaches for network trimming. In pathway reconstruction and the inference of context-specific networks, we compared our method with three popular tools: PL, OI2, and DOMINO. Hub proteins may dominate the inferred network with unrelated interactions. The prize-collecting Steiner Forest algorithm penalizes hubs based on the number of interactions. Similarly, the flux calculation in pyPARAGON is a countermeasure against the curse of hubs beyond scoring interactions. OI2 and pyPARAGON work better at predicting interactions. Regarding the identification of associated genes, our tool outperformed the other tools. In the PL algorithm, highly connected nodes further diminish the shortest paths between seed nodes. OI2 early terminates the propagation of the seed nodes in a large reference network. However, the PageRank algorithm in pyPARAGON propagates the seed nodes before network inference, independent of GGN. Thus, pyPARAGON optimizes the inference of interactions and the propagation of seed nodes in the network.

In conclusion, we released pyPARAGON as a novel tool, which infers context-specific networks by using graphlets and network propagation. pyPARAGON can infer a network from the omic datasets and potentially predict context-specific biomarkers, drugs, and therapeutic targets. For downstream analysis, communities in the network can potentially identify mechanistic molecular relations in complex and rare diseases. Here, pyPARAGAON integrated bulk omic data for static tumor-specific network models. The next version of pyPARAGON will be an extension that incorporates omic data at the single-cell level to elucidate cell-type specific interactions.

**Key Points**

- pyPARAGON combines graphlets with network propagation using the PPR algorithm. This is followed by interaction selection based on edge flux calculation, effectively addressing challenges in network modeling such as the inclusion of false positive proteins/genes and interactions, as well as accounting for the dominance of hubs and obscure context-specific relationships.
- pyPARAGON is an open-source method that offers easy accessibility and can be run in local environments. This feature provides a significant advantage for research groups interested in omic data integration.
- In constructing cancer signaling pathways and identifying cancer driver networks, pyPARAGON outperforms other state-of-the-art approaches in terms of node propagation and edge inference.
- We found that network trimming through graphlets plays a crucial role in improving the performance of network inference.
- pyPARAGON can construct tumor-specific networks, revealing hidden commonalities across tumors.

## Supplementary Material

Supplementary_Figures_final_bbae399

Supplementary_Tables_final_bbae399

Supplementary_Methods_final_bbae399

## Data Availability

The results shown here are in whole or part based upon data generated by the TCGA Research Network: https://www.cancer.gov/tcga. The input data and source codes to reproduce the results are available at https://github.com/netlab-ku/pyPARAGON. The list of cancer driver genes was retrieved from Integrative Onco Genomics (intOGen): https://www.intogen.org. The reference PPI networks, HIPPIE (v2.2 and v2.3), and ConsensusPathDB were retrieved from https://cbdm-01.zdv.uni-mainz.de/∼mschaefer/hippie/download.php and http://cpdb.molgen.mpg.de, respectively.
