# Supplementary material for: Unveiling hidden connections in omics data via pyPARAGON: an integrative hybrid approach for disease network construction
Source: Brief Bioinform. 2024 Aug 20;25(5):bbae399. doi: 10.1093/bib/bbae399 (PMC11334722; doi:10.1093/bib/bbae399)
Supplement: Supplementary_Figures_final_bbae399 [file supplementary_figures_final_bbae399.docx]

**Supplementary Figures**


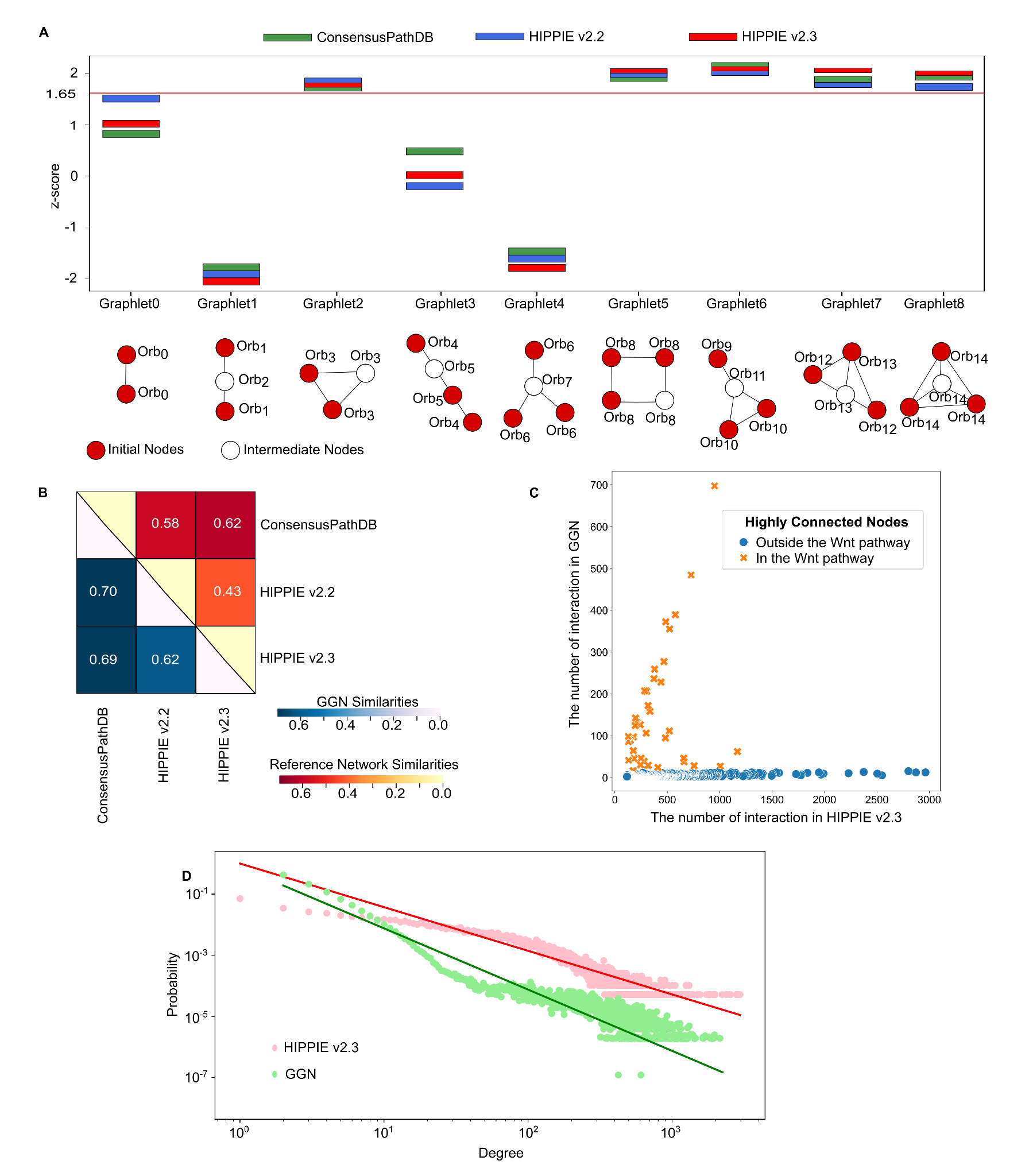


**Figure S1:** **Graphlet-guided networks (GGN) optimize reference networks**. **a.** Graphlets composed of 2, 3, and 4 nodes are constructed with initial nodes (red circle) coming from the given input and intermediate nodes (white circle). Intermediate nodes are the ones that have the highest connections to the seed nodes in the corresponding graphlet. We compared the frequencies of graphlets on different reference interactomes with their 100 permuted networks. Despite having different network sizes and properties, ConsensusPathDB (green), HIPPIE v2.2 (blue), and HIPPIE v2.3 (red) have similar graphlet motifs, such as Graphlets 2, 5, 6, 7, and 8 for signaling pathways in NetPath (p<0.05). **b.** The heatmap with the gradual color change highlighted the network similarities between reference networks (red) and GGNs (blue). The Jaccard Similarity Index was determined by dividing the number of common interactions by the number of merged interactions. The top-right section depicts network similarities, whereas the bottom-left section depicts average GGN similarities retrieved with the same initial nodes from NetPath. The construction of GGN results in more comparable and optimized networks. **c.** The remaining highly connected nodes in specific GGN for the Wnt pathway**.** pyPARAGON uses half of the nodes in the Wnt pathway during the reconstruction of the GGN. 1229 of 3887 highly connected nodes in HIPPIE are still part of GGN. However, just 47 nodes from the remaining highly connected nodes participate in the Wnt pathway. Their interactions are maintained in GGN, depending on the initial node set. On the other hand, irrelevant highly connected nodes lost most of their interactions **d.** pink and green distributions depict the degree probabilities of HIPPIE v2.3 and GGN as a scale-free network. The slopes of the graphs demonstrate experimental degree exponent (γ_HIPPIE_=1.45 R^2^_HIPPIE_=0.88, and γ_GGNs_=2.40, R^2^_GGNs_=0.84). While strongly following the power law, GGN was constructed with nodes at various degrees to avoid the noise of highly connected nodes by reducing their irrelevant interactions. GGN retains scale-free network features, as seen in biological networks.


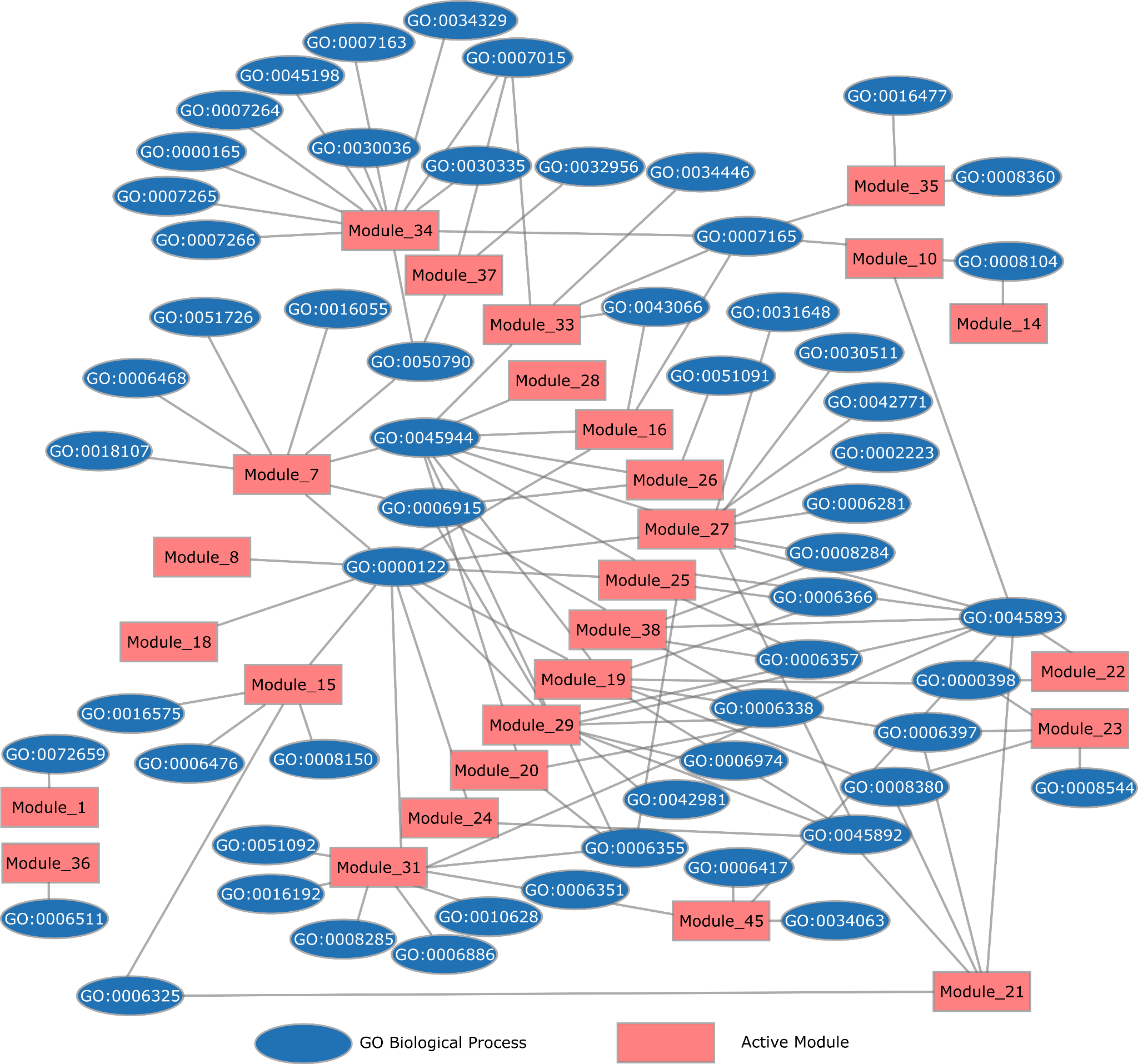


**Figure S2: The association of active modules and biological processes:** Pale red and blue represent active modules and biological processes, respectively. An active module can be associated with multiple biological processes, or a biological process can be related to multiple active modules.


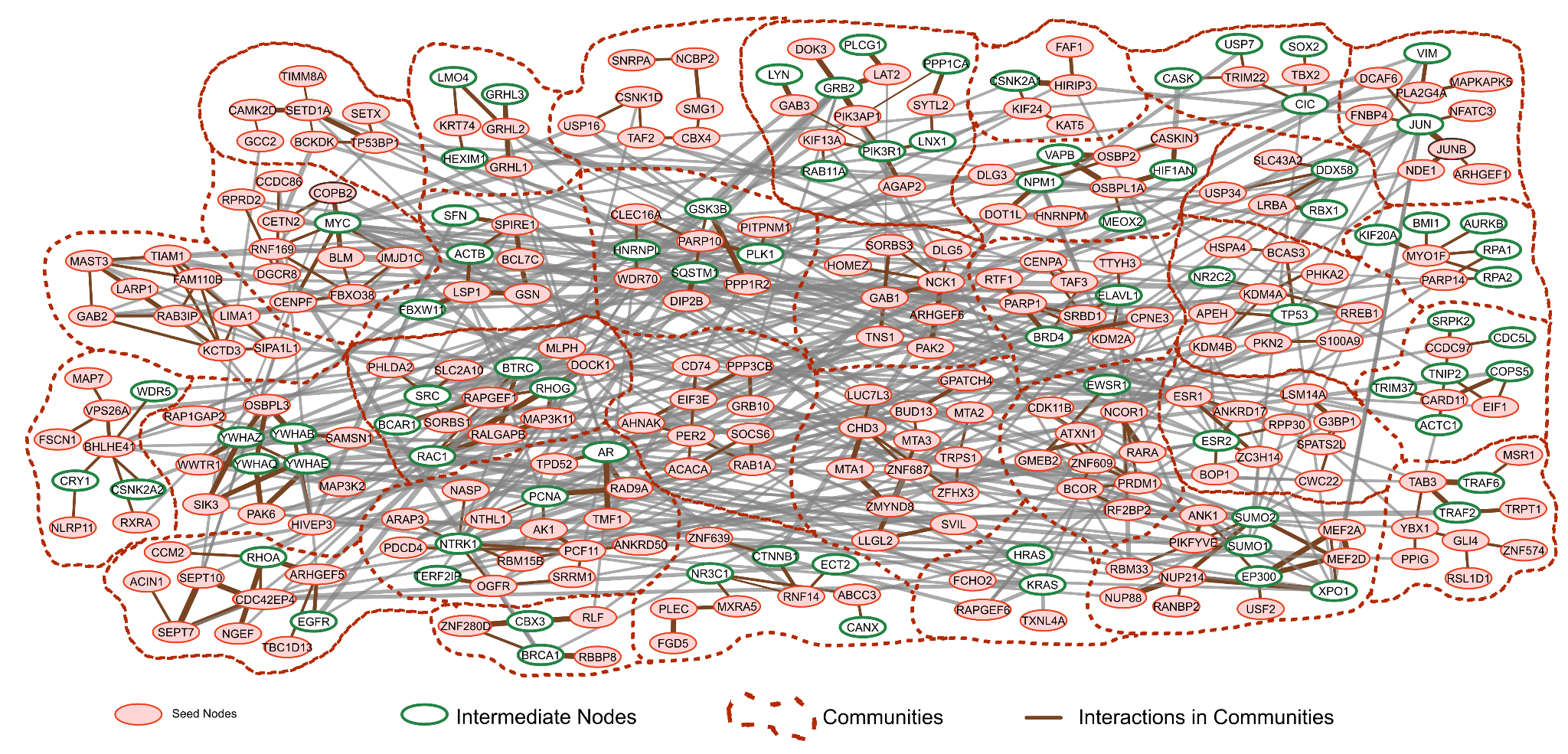


**Figure S3: Patient (TCGA-A8-A079)-specific networks and their modules:** Seed and intermediate nodes are demonstrated with pink cycle and green circle, respectively. Modules, and communities in networks are bordered with dashed red lines. Only interactions between module participants are drawn with brown links. The remaining interactions in the network demonstrate crosstalks among modules and are represented with gray color. The size of interactions between 0.49 and 1.0 is correlated with the confidence score of interactions.


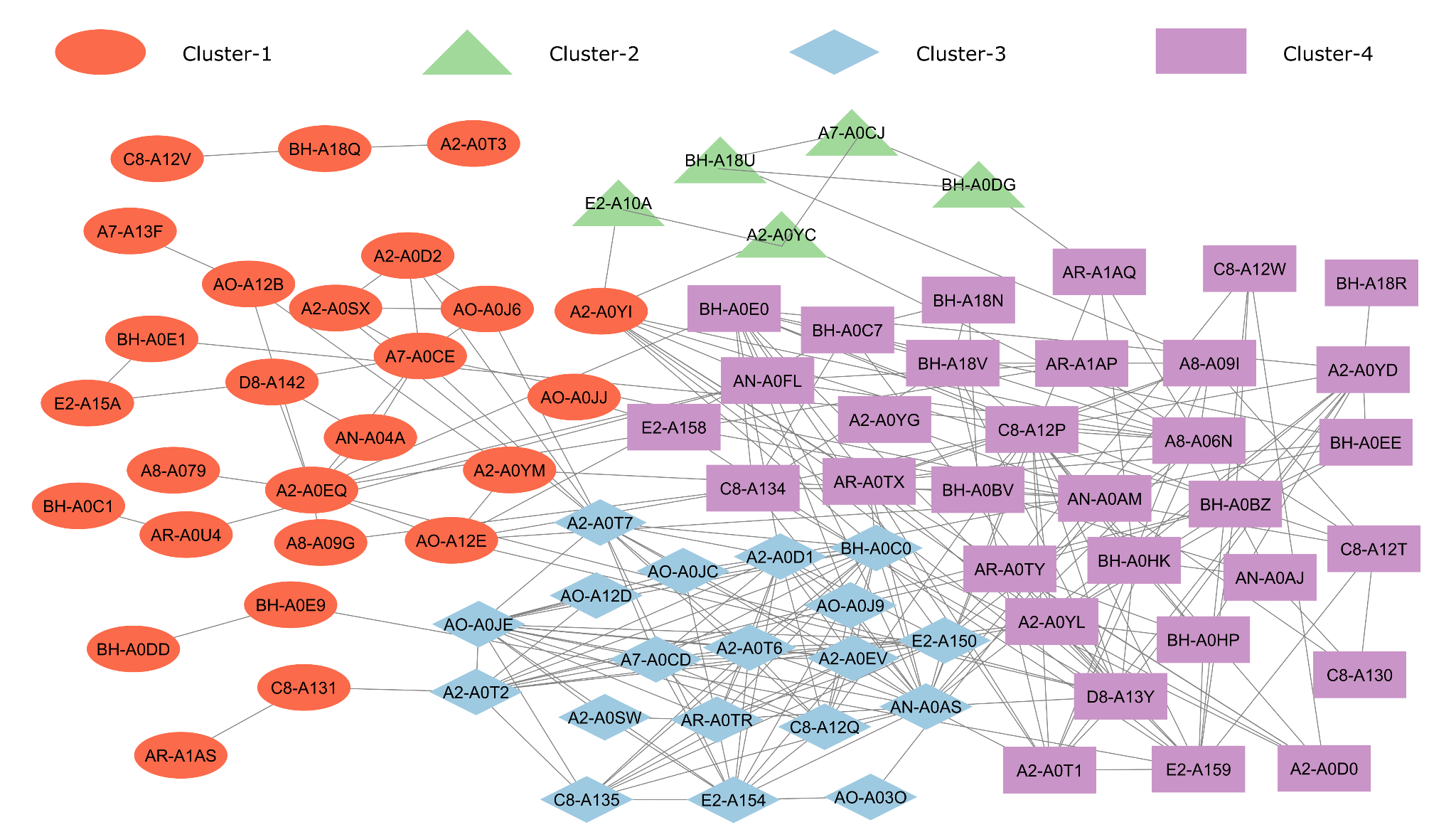


**Figure S4: Patient similarity network.** The similarities of 105 patients were calculated through a cosine similarity score of meaningful biological processes between patient pairs. In the similarity network, we illustrated interactions between patients with similarity scores greater than 0.5 (82 patients, 262 interactions). In the similarity network, we displayed interactions between patients with similarity scores greater than 0.5. (82 patients, 262 interactions). Using the t-SNE algorithm and agglomerative clustering, we divided the patients into four groups based on biological processes. Cluster-1 (26 patients) was represented by red ellipses, Cluster-2 (5 patients) by green triangles, Cluster-3 (19 patients) by blue diamonds, and Cluster-4 (32 patients) by purple rectangles. Most patients in Cluster-2 do not have obvious similarities in patient pairs, while most in Cluster-3 and Cluster-4 do have higher similarities and more interactions in the patient similarity network.

**
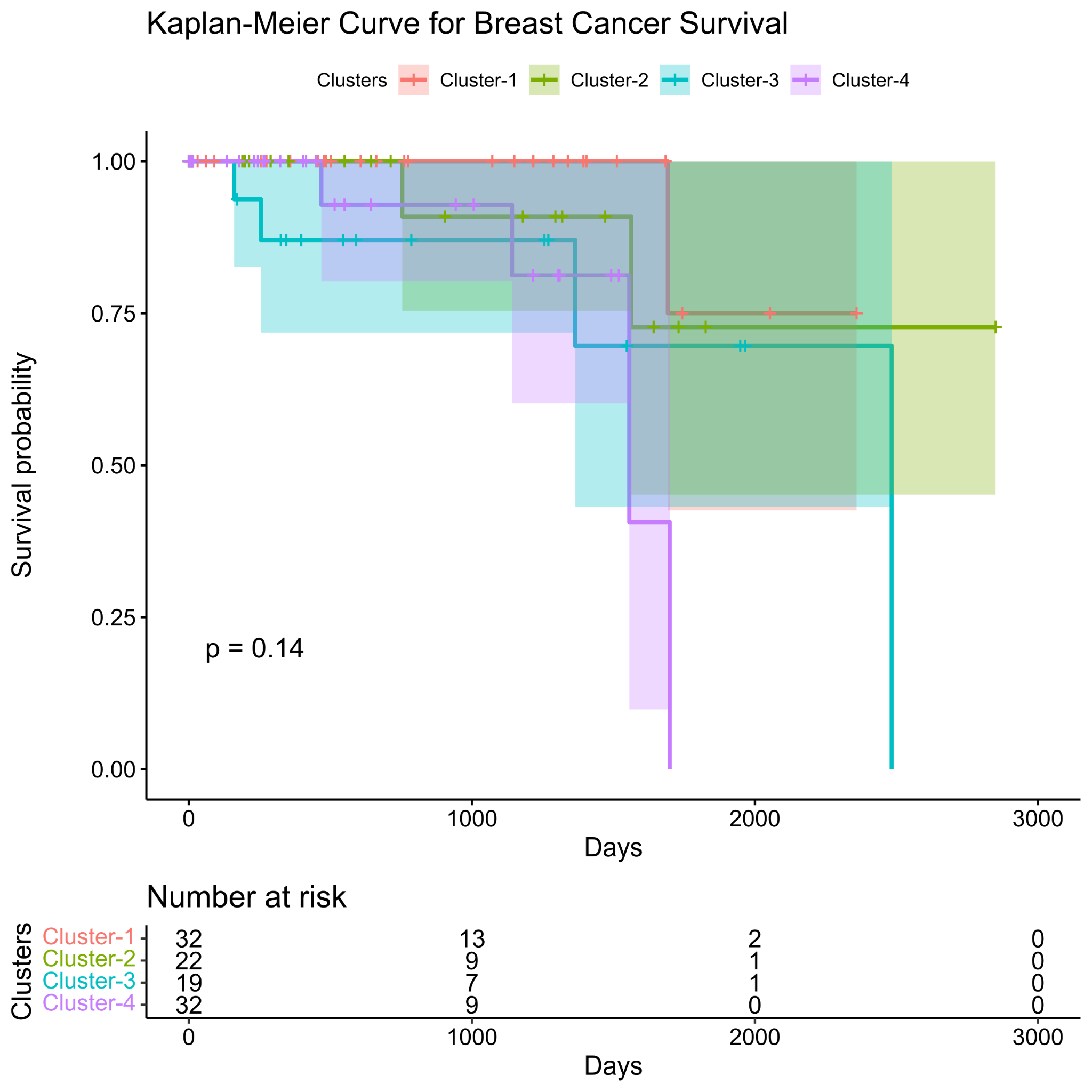
**

**Figure S5: The survival probabilities of clusters.** The Kaplan-Meier (K-M) curve analysis of clustered breast cancer showed that there was not any major difference in survival probabilities or risks among the four clusters. However, the lowest survival probability seems to be in cluster-4; the highest survival probability is in cluster-1.


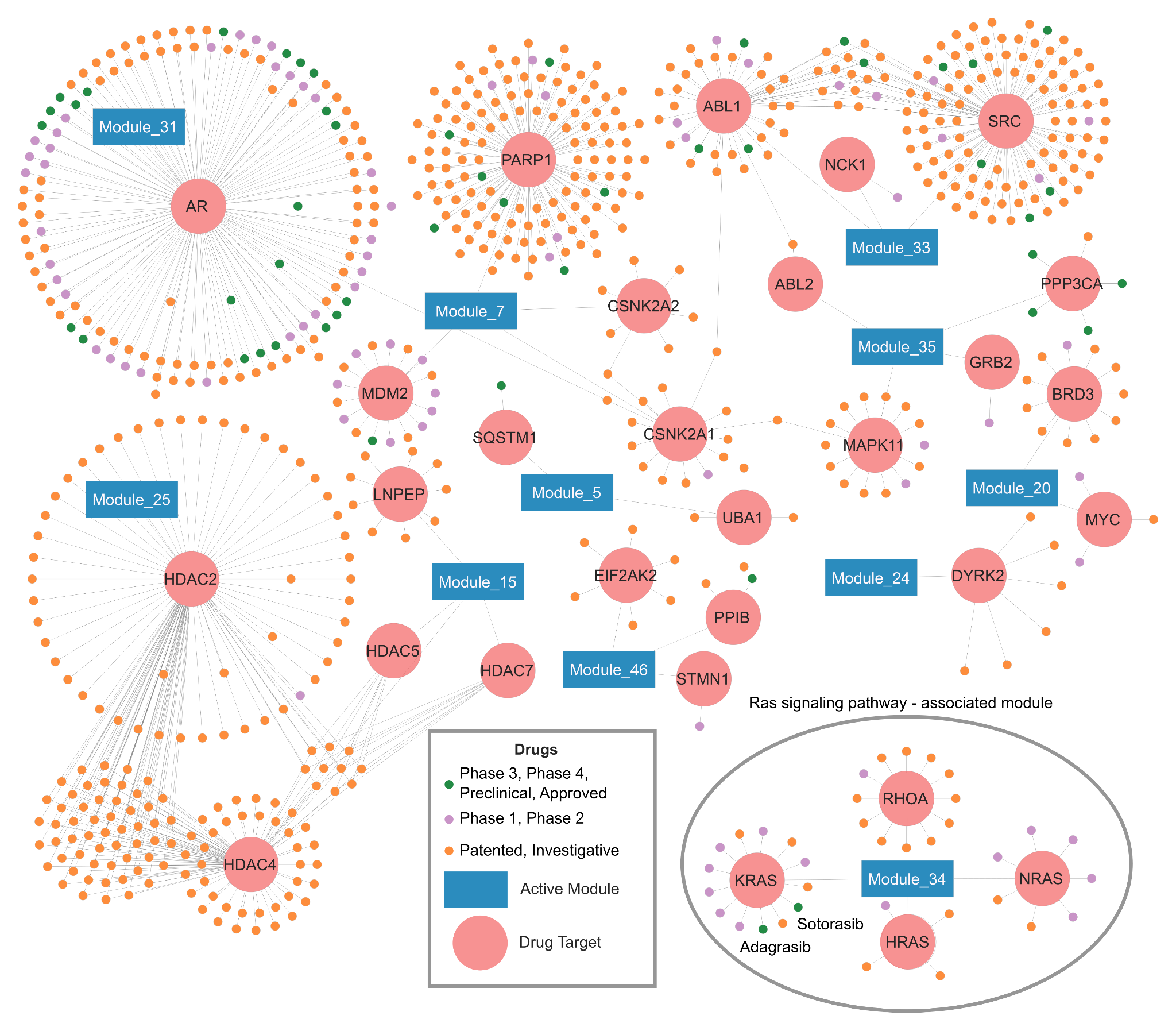


**Figure S6: Drug-module interaction network of a patient (TCGA-A2-A9YD)**. Drugs are shown in three colors corresponding to three categories: drugs in phase 3, 4, or preclinical stage and authorized drugs in green; drugs in phase 2 or 3 in purple; and patented and investigational drugs in pink.
