# Supplementary material for: Unveiling hidden connections in omics data via pyPARAGON: an integrative hybrid approach for disease network construction
Source: Brief Bioinform. 2024 Aug 20;25(5):bbae399. doi: 10.1093/bib/bbae399 (PMC11334722; doi:10.1093/bib/bbae399)
Supplement: Supplementary_Tables_final_bbae399 [file supplementary_tables_final_bbae399.docx]

**Supplementary Tables**

**Table 1:** Topological Features of Reference Networks

|  | ConsensusPathDB | GGNs from ConsensusPathDB | HIPPIE v2.2 | GGNs from HIPPIE v2.2 | HIPPIE v2.3 | GGNs from HIPPIE v2.3 |
| --- | --- | --- | --- | --- | --- | --- |
| The number of nodes | 18 178 | 2240 | 15 861 | 1824 | 19 437 | 2806 |
| The number of edges | 516 211 | 8916 | 345 770 | 7138 | 774 449 | 11579 |
| The number of highly connected nodes (20% of nodes with the highest degree) | 3636 | 2240 | 3172 | 1820 | 3887 | 2405 |
| Average node degree | 56.8 | 7.26 | 43.6 | 7.27 | 79.6 | 7.52 |
| Diameter | 8 | 4 | 8 | 4 | 7 | 4 |

**Table 2:** Positive and negative counts in HIPPIE v2.3

| **Pathway** | **Negative Edges** | **Negative Nodes** | **Positive Edges** | **Positive Nodes** |
| --- | --- | --- | --- | --- |
| Alpha6Beta4Integrin | 774223 | 19371 | 225 | 33 |
| AndrogenReceptor | 773965 | 19274 | 483 | 82 |
| BCR | 773972 | 19301 | 476 | 68 |
| BDNF | 774301 | 19366 | 147 | 36 |
| EGFR1 | 772941 | 19206 | 1507 | 116 |
| IL2 | 774191 | 19370 | 257 | 34 |
| IL3 | 774265 | 19367 | 183 | 35 |
| IL4 | 774269 | 19380 | 179 | 28 |
| IL6 | 774293 | 19385 | 155 | 26 |
| KitReceptor | 774228 | 19363 | 220 | 37 |
| Leptin | 774308 | 19382 | 140 | 28 |
| Notch | 774178 | 19363 | 270 | 37 |
| Prolactin | 774240 | 19369 | 208 | 34 |
| RANKL | 774303 | 19380 | 145 | 28 |
| TCR | 773933 | 19284 | 515 | 76 |
| TGFbetaReceptor | 773564 | 19230 | 884 | 104 |
| TNFalpha | 773516 | 19200 | 932 | 118 |
| Wnt | 774007 | 19331 | 441 | 53 |

**Table 3:** Performance metrics of cancer-specific network

| **Tools** | **Cancer Types** | **The Number of Nodes** | **The Number  of Edges** | **Recall** | **Precision** | **F1 Score** | **AUPRC** |
| --- | --- | --- | --- | --- | --- | --- | --- |
| PathLinker | BLCA | 351 | 795 | 0.180 | 0.082 | 0.113 | 0.009 |
| PathLinker | BRCA | 330 | 762 | 0.212 | 0.128 | 0.160 | 0.016 |
| PathLinker | ESCA | 306 | 721 | 0.207 | 0.098 | 0.133 | 0.012 |
| PathLinker | HNSC | 323 | 729 | 0.231 | 0.084 | 0.123 | 0.012 |
| PathLinker | LUSC | 285 | 598 | 0.176 | 0.053 | 0.081 | 0.006 |
| PathLinker | PAAD | 330 | 688 | 0.126 | 0.037 | 0.057 | 0.003 |
| PathLinker | PRAD | 309 | 693 | 0.194 | 0.106 | 0.137 | 0.012 |
| PathLinker | UCEC | 281 | 605 | 0.228 | 0.108 | 0.146 | 0.015 |
| pyPARAGON | BLCA | 521 | 1709 | 0.317 | 0.112 | 0.165 | 0.021 |
| pyPARAGON | BRCA | 540 | 1711 | 0.263 | 0.126 | 0.170 | 0.020 |
| pyPARAGON | ESCA | 587 | 1688 | 0.286 | 0.094 | 0.142 | 0.016 |
| pyPARAGON | HNSC | 549 | 1585 | 0.333 | 0.113 | 0.169 | 0.023 |
| pyPARAGON | LUSC | 614 | 1687 | 0.271 | 0.083 | 0.127 | 0.013 |
| pyPARAGON | PAAD | 499 | 1397 | 0.200 | 0.084 | 0.118 | 0.010 |
| pyPARAGON | PRAD | 557 | 1683 | 0.312 | 0.125 | 0.179 | 0.023 |
| pyPARAGON | UCEC | 543 | 1679 | 0.251 | 0.112 | 0.155 | 0.017 |
| Omics Integrator2 | BLCA | 426 | 794 | 0.085 | 0.026 | 0.040 | 0.001 |
| Omics Integrator2 | BRCA | 438 | 793 | 0.146 | 0.054 | 0.079 | 0.005 |
| Omics Integrator2 | ESCA | 458 | 814 | 0.138 | 0.038 | 0.059 | 0.003 |
| Omics Integrator2 | HNSC | 463 | 842 | 0.112 | 0.028 | 0.045 | 0.002 |
| Omics Integrator2 | LUSC | 470 | 798 | 0.920 | 0.024 | 0.047 | 0.013 |
| Omics Integrator2 | PAAD | 432 | 771 | 0.105 | 0.020 | 0.034 | 0.001 |
| Omics Integrator2 | PRAD | 451 | 788 | 0.083 | 0.024 | 0.037 | 0.001 |
| Omics Integrator2 | UCEC | 442 | 788 | 0.068 | 0.015 | 0.025 | 0.001 |
| DOMINO | BLCA | 366 | 825 | 0.178 | 0.063 | 0.093 | 0.007 |
| DOMINO | BRCA | 442 | 894 | 0.158 | 0.024 | 0.042 | 0.002 |
| DOMINO | ESCA | 392 | 940 | 0.137 | 0.039 | 0.060 | 0.003 |
| DOMINO | HNSC | 340 | 810 | 0.188 | 0.069 | 0.101 | 0.008 |
| DOMINO | LUSC | 468 | 1024 | 0.147 | 0.036 | 0.058 | 0.003 |
| DOMINO | PAAD | 448 | 962 | 0.144 | 0.028 | 0.047 | 0.002 |
| DOMINO | PRAD | 454 | 983 | 0.178 | 0.037 | 0.061 | 0.004 |
| DOMINO | UCEC | 435 | 894 | 0.150 | 0.046 | 0.071 | 0.004 |

**Table 4:** Frequently seen biological processes in clusters

| **GO ID** | **The number of patients** | **Name** | **Cluster** |
| --- | --- | --- | --- |
| GO:0006511 | 21 | ubiquitin-dependent protein catabolic process | Cluster1 |
| GO:0006605 | 20 | protein targeting | Cluster1 |
| GO:0051056 | 16 | regulation of small GTPase mediated signal transduction | Cluster1 |
| GO:0031146 | 16 | SCF-dependent proteasomal ubiquitin-dependent protein catabolic process | Cluster1 |
| GO:0015031 | 15 | protein transport | Cluster1 |
| GO:0010628 | 15 | positive regulation of gene expression | Cluster1 |
| GO:0008150 | 13 | biological_process | Cluster1 |
| GO:0042659 | 13 | regulation of cell fate specification | Cluster1 |
| GO:0000281 | 13 | mitotic cytokinesis | Cluster1 |
| GO:2000736 | 13 | regulation of stem cell differentiation | Cluster1 |
| GO:0030154 | 12 | cell differentiation | Cluster1 |
| GO:0045087 | 12 | innate immune response | Cluster1 |
| GO:0007399 | 12 | nervous system development | Cluster1 |
| GO:0070936 | 12 | protein K48-linked ubiquitination | Cluster1 |
| GO:0000165 | 12 | MAPK cascade | Cluster1 |
| GO:0007155 | 12 | cell adhesion | Cluster1 |
| GO:0032956 | 11 | regulation of actin cytoskeleton organization | Cluster1 |
| GO:0009410 | 11 | response to xenobiotic stimulus | Cluster1 |
| GO:0006886 | 10 | intracellular protein transport | Cluster1 |
| GO:0007010 | 10 | cytoskeleton organization | Cluster1 |
| GO:0000281 | 13 | mitotic cytokinesis | Cluster2 |
| GO:0015031 | 12 | protein transport | Cluster2 |
| GO:0030154 | 12 | cell differentiation | Cluster2 |
| GO:0051123 | 11 | RNA polymerase II pre-initiation complex assembly | Cluster2 |
| GO:0060261 | 11 | positive regulation of transcription initiation by RNA polymerase II | Cluster2 |
| GO:0043123 | 11 | positive regulation of I-kappaB kinase/NF-kappaB signaling | Cluster2 |
| GO:0018105 | 11 | peptidyl-serine phosphorylation | Cluster2 |
| GO:0000165 | 10 | MAPK cascade | Cluster2 |
| GO:0007010 | 10 | cytoskeleton organization | Cluster2 |
| GO:0008150 | 10 | biological_process | Cluster2 |
| GO:0043065 | 10 | positive regulation of apoptotic process | Cluster2 |
| GO:0008285 | 9 | negative regulation of cell population proliferation | Cluster2 |
| GO:0050821 | 9 | protein stabilization | Cluster2 |
| GO:0006511 | 8 | ubiquitin-dependent protein catabolic process | Cluster2 |
| GO:0032968 | 8 | positive regulation of transcription elongation by RNA polymerase II | Cluster2 |
| GO:0032922 | 8 | circadian regulation of gene expression | Cluster2 |
| GO:0007266 | 7 | Rho protein signal transduction | Cluster2 |
| GO:0016055 | 7 | Wnt signaling pathway | Cluster2 |
| GO:0006897 | 7 | endocytosis | Cluster2 |
| GO:0007155 | 7 | cell adhesion | Cluster2 |
| GO:2000045 | 19 | regulation of G1/S transition of mitotic cell cycle | Cluster3 |
| GO:2000819 | 19 | regulation of nucleotide-excision repair | Cluster3 |
| GO:0030071 | 19 | regulation of mitotic metaphase/anaphase transition | Cluster3 |
| GO:2000781 | 19 | positive regulation of double-strand break repair | Cluster3 |
| GO:0070316 | 19 | regulation of G0 to G1 transition | Cluster3 |
| GO:1902459 | 14 | positive regulation of stem cell population maintenance | Cluster3 |
| GO:0007399 | 14 | nervous system development | Cluster3 |
| GO:0045663 | 13 | positive regulation of myoblast differentiation | Cluster3 |
| GO:0045597 | 13 | positive regulation of cell differentiation | Cluster3 |
| GO:0045582 | 12 | positive regulation of T cell differentiation | Cluster3 |
| GO:0006897 | 11 | endocytosis | Cluster3 |
| GO:0006605 | 10 | protein targeting | Cluster3 |
| GO:0045596 | 10 | negative regulation of cell differentiation | Cluster3 |
| GO:0016055 | 9 | Wnt signaling pathway | Cluster3 |
| GO:0006913 | 9 | nucleocytoplasmic transport | Cluster3 |
| GO:0000165 | 9 | MAPK cascade | Cluster3 |
| GO:0006337 | 8 | nucleosome disassembly | Cluster3 |
| GO:0015031 | 8 | protein transport | Cluster3 |
| GO:0006511 | 8 | ubiquitin-dependent protein catabolic process | Cluster3 |
| GO:0030154 | 8 | cell differentiation | Cluster3 |
| GO:0032956 | 23 | regulation of actin cytoskeleton organization | Cluster4 |
| GO:0007155 | 19 | cell adhesion | Cluster4 |
| GO:0008150 | 18 | biological_process | Cluster4 |
| GO:0030154 | 18 | cell differentiation | Cluster4 |
| GO:0030865 | 17 | cortical cytoskeleton organization | Cluster4 |
| GO:0000165 | 16 | MAPK cascade | Cluster4 |
| GO:0034063 | 16 | stress granule assembly | Cluster4 |
| GO:0006605 | 16 | protein targeting | Cluster4 |
| GO:0006897 | 14 | endocytosis | Cluster4 |
| GO:0015031 | 14 | protein transport | Cluster4 |
| GO:0006417 | 13 | regulation of translation | Cluster4 |
| GO:0007266 | 13 | Rho protein signal transduction | Cluster4 |
| GO:0043065 | 12 | positive regulation of apoptotic process | Cluster4 |
| GO:0051496 | 12 | positive regulation of stress fiber assembly | Cluster4 |
| GO:0018107 | 12 | peptidyl-threonine phosphorylation | Cluster4 |
| GO:0046777 | 12 | protein autophosphorylation | Cluster4 |
| GO:0007010 | 12 | cytoskeleton organization | Cluster4 |
| GO:0032968 | 11 | positive regulation of transcription elongation by RNA polymerase II | Cluster4 |
| GO:0051056 | 11 | regulation of small GTPase mediated signal transduction | Cluster4 |
| GO:0008285 | 11 | negative regulation of cell population proliferation | Cluster4 |

**Table 5:** TFs regulating CDKs and their frequencies in clusters

| **TFs** | **cluster4** | **cluster3** | **cluster2** | **cluster1** |
| --- | --- | --- | --- | --- |
| **TP53** | 32 | 18 | 22 | 30 |
| **ESR2** | 32 | 19 | 22 | 31 |
| **ESR1** | 32 | 17 | 22 | 32 |
| **MYC** | 31 | 19 | 22 | 32 |
| **JUN** | 31 | 18 | 18 | 29 |
| **NR3C1** | 30 | 17 | 19 | 29 |
| **AR** | 30 | 17 | 21 | 28 |
| **HDAC1** | 30 | 18 | 19 | 28 |
| **EP300** | 29 | 17 | 22 | 30 |
| **HDAC7** | 25 | 12 | 9 | 14 |
| **HDAC2** | 23 | 10 | 16 | 19 |
| **YBX1** | 21 | 11 | 12 | 16 |
| **COPS5** | 21 | 11 | 16 | 20 |
| **SMARCA4** | 19 | 13 | 6 | 10 |
| **RB1CC1** | 19 | 12 | 13 | 12 |
| **ABL1** | 18 | 13 | 10 | 13 |
| **DNMT3A** | 17 | 10 | 6 | 14 |
| **CREBBP** | 17 | 10 | 12 | 11 |
| **EZH2** | 16 | 11 | 7 | 10 |
| **RB1** | 16 | 7 | 9 | 8 |
| **DMAP1** | 13 | 7 | 8 | 11 |
| **PARP1** | 13 | 9 | 11 | 13 |
| **BRCA1** | 13 | 12 | 17 | 24 |

**Table S6:** FDA-approved drugs targeting TFs regulating CDKs

| **TFs** | **DrugID** |
| --- | --- |
| ABL1 | 'D01BYB', 'D0OB0F', 'D0H0EQ', 'D03MNN' |
| AR | 'D0S7LG', 'D0V9BD', 'D08TEJ', 'D06AEO', 'D0DV6D', 'D09NNA', 'D0QK5X', 'D0SC8F', 'D0R7JT', 'D0B2WJ', 'D0L2LS', 'D0Y0SW', 'D0BC2E', 'D00YWP', 'D0SN9T', 'D0U3GL', 'D0K0EK', 'D06XMU' |
| ESR1 | 'D06LOQ', 'D0JY8T', 'D0N6YV', 'D0CT9Y', 'D0I5WB', 'D0A2RG', 'D01ICU', 'D0X1EZ', 'D09IPV', 'D09ZQN', 'D0Y2NE', 'D0R6RE', 'D08QMX', 'D0T7ZQ', 'D0U0XD', 'D07VBA', 'D0Z1FX', 'D0M8PD', 'D00ZFP', 'D03IUY', 'D06NXY', 'D0JO8Z', 'D0JO7Y', 'D0M5RF', 'D09NMD', 'D0W9GA', 'D0J1ML', 'D0Z5OE', 'D0V2JK', 'D02CTS', 'D0S1UW', 'D0C4NY', 'D04UZT', 'D01XBA', 'D07KSG', 'D04VFJ' |
| ESR2 | 'D06LOQ', 'D0T0LU', 'D0M8PD', 'D03XOC' |
| EZH2 | 'D00EQL' |
| HDAC1 | 'D0E3SH', 'D0L7LC', 'D0E7PQ' |
| NR3C1 | 'D0CW1P', 'D06CWH', 'D03SXE', 'D0Y7IU', 'D09WYX', 'D0IT2G', 'D0NA8U', 'D0FM2P', 'D02QJH', 'D0P0HT', 'D0LC6K', 'D0Y7JU', 'D05RXI', 'D0KR5B', 'D0I5DS', 'D08PIQ', 'D0Z4EI', 'D03HYX', 'D0D1SG', 'D0IL7L', 'D03BLF' |
| PARP1 | 'D09LSX', 'D06NVJ', 'D0DN0W' |
