# Supplementary material for: Unveiling hidden connections in omics data via pyPARAGON: an integrative hybrid approach for disease network construction
Source: Brief Bioinform. 2024 Aug 20;25(5):bbae399. doi: 10.1093/bib/bbae399 (PMC11334722; doi:10.1093/bib/bbae399)
Supplement: Supplementary_Methods_final_bbae399 [file supplementary_methods_final_bbae399.docx]

# Supplementary Methods

## **Interactomes and Datasets**

Reference networks are interactomes that serve as the basis for network propagation and reconstruction. These networks can be retrieved from interactome databases (e.g., HIPPIE, String) or constructed with prior knowledge, experimental data, or computational predictions. We separately used interactomes; HIPPIE v2.2 (15 861 nodes, 345 770 edges), HIPPIE v2.3 (19 437 nodes, 774 449 edges), and ConsensusPathDB v35 (18 178 nodes, 516 211) [[1,2]](https://paperpile.com/c/GQknHk/x27S+ZJxt). Interactomes are separately used as a reference network, ***G(V, E, c(e))*** where ***V*** is the node set, ***E*** is the undirected edge set, and ***c(e)*** is the weight of an edge. HIPPIE interactomes are weighted by confidence scores in the interactions, ***0≤c(e)≤1***. Because ConsensusPathDB provides an unweighted interactome, ***c(e)*** is set to be ***1.0*.**

As a benchmark to reconstruct pathways, we utilized 18 cancer signaling pathways in NetPath, each composed of more than 50 proteins [[3]](https://paperpile.com/c/GQknHk/umH3). To infer networks particular to each kind, we compiled seed node sets for different cancer types. We selected the 300 genes that were most frequently mutated in each cancer type, based on a total patient cohort of 3,759. The mutation dataset covers various cancer genomics projects, including TCGA and GENIE [[4]](https://paperpile.com/c/GQknHk/B6Pb). IntOGen is composed of 3333 driver mutations in 568 genes across 66 cancer types. After selecting cancer types with more than 50 driver genes that are specified in TCGA and GENIE, our analysis is limited to eight cancer types: bladder urothelial carcinoma (BLCA), breast invasive carcinoma (BRCA), esophageal carcinoma (ESCA), head and neck squamous cell carcinoma (HNSC), lung squamous cell carcinoma (LUSC), pancreatic adenocarcinoma (PAAD), prostate adenocarcinoma (PRAD), and uterine corpus endometrial carcinoma (UCEC). Therefore, we retrieved 1138 driver mutations in 279 genes belonging to the eight cancer types in IntOGen (provided in [pyPARAGON/Supplementaries](https://github.com/netlab-ku/pyPARAGON/tree/main/Supplementaries) folder on GitHub) [[5]](https://paperpile.com/c/GQknHk/77NX).

We utilized phosphoproteomics data for 105 breast tumors and three healthy samples [[6]](https://paperpile.com/c/GQknHk/RN3o). In network inference, seed nodes (the initial node set) are selected based on their demonstrated relevance or significant alterations in the current experimental context (e.g., differentially expressed genes, significantly active transcription factors, mutated proteins, etc.). To select the seed nodes, we first selected phosphosites with a standard deviation greater than 0.5 and present in more than 50% of all samples. Next, we separately categorized phosphoproteins by assessing each patient’s phosphoproteins according to two criteria: (1) having |log2(FC)| > 2.0, and (2) highly or less phosphorylated in the Gaussian Mixture Model (GMM) [[7]](https://paperpile.com/c/GQknHk/LBH1). In GMM, we split phosphoproteomics into three divisions: highly, less, or normally phosphorylated proteins. We ran the model 100 times randomly for each phosphoproteomics and chose highly or less phosphorylated proteins in 95% of the models. Using the unit-variance scaling method on LFC, we gave differential phosphoprotein scores between 0.5 and 1[[8]](https://paperpile.com/c/GQknHk/uL30). We utilized biological processes retrieved from gene ontology, pathways from KEGG, subcellular localization from the human proteome atlas, transcription factors and targets from TRRUST.v2, and drug information from the therapeutic target database to annotate network modules [[9–13]](https://paperpile.com/c/GQknHk/XHCZ+RCrm+PAbX+NGhk+Y0lL). The network module may be described as a distinct subset of nodes that exhibit a greater level of internal connectivity than nodes outside the subset. The annotation of modules elucidates the functional components of the network, facilitating biological interpretation.

## **Performance Assessment of Network Inference Tools**

Network inference tools construct context-specific networks, like gene regulatory networks or protein-protein interaction networks, from experimental data or integrated databases. pyPARAGON takes a list of seed genes/proteins (initial nodes) as input, which are specific to the biological context of interest. Seeds can be obtained from but not limited to omics experiments, drug perturbation analysis, or disease-associated proteins. The inferred network may contain nodes and interactions that do not relate to the experimental context, known as false positives. Graphlets help in reducing the load of possible false positives, while network propagation via PPR scores all other proteins in the reference interactome by orienting them from the given seed nodes. This hybrid approach, on the one hand, trims the reference interactome to the most relevant interactions by constructing a GGN, on the other hand, it quantifies the importance of other proteins based on a given seed list.

## Network inference tools

We compared pyPARAGON with Omics Integrator 2 (OI2) [[14]](https://paperpile.com/c/GQknHk/Cfo7), PathLinker 1.4.3 (PL) [[15,16]](https://paperpile.com/c/GQknHk/SnyM+wveS), and DOMINO [[17]](https://paperpile.com/c/GQknHk/xbir) by reconstructing pathways in NetPath and inferring specific cancer networks. Network inference tools use a reference network (***G(V, E, c(e))***) and seed node sets (***X ∈ V***).

## Omics Integrator 2

OI2 implements the prize-collecting Steiner Forest algorithm [[14]](https://paperpile.com/c/GQknHk/Cfo7). The objective function of OI2 combines confidence scores of edges (***c(e)***) and penalties of edges calculated with node degrees. Based on the degree in the reference network, the negative weights are given to nodes, as stated in formula 1:

$p'(v) = \beta. p(v) -\mu. deg(v)$ (1)

where ***v*** is the node in the given reference network, ***β*** controls the relative weight of the node prizes to calibrate the effect of terminal nodes, ***μ*** affects the penalty based on the degree of a node to calibrate the impact of hub nodes, and ***deg(v )*** is the number of interactions.

OI2 infers the subnetwork ***F(V_F_, E_F_)***, where ***V_F_ ∈ V, E_F_ ∈ E***, to minimize the prize function by using formula 2;

$f^{'}\left( F \right)= \sum_{v \notin V_{f}} p^{'}\left( v \right) +\sum_{e \in E_{f}} cost\left( e \right) +\omega. \kappa$ (2)

where ***κ*** is the number of connected components, ***ω*** controls the cost of adding a tree to the solution network, and ***cost(e)*** is the cost of the given edge. The cost of each edge is calculated by extracting the confidence score of the edge from 1.5.

## PathLinker

PL computes the ***k***-highest scoring short paths between seed nodes without a loop in the reference network. The path score, ***W***, is the product of the edge weights along the path [[15,16]](https://paperpile.com/c/GQknHk/SnyM+wveS). PL calculates the cost of a path with the formula 3:

$C_{uv}=\left\{ \begin{aligned} -log \left( W_{uv} \right) if u,v \epsilon V \left\{ s,t \right\} \\ 0 if u = s or v=t \end{aligned} \right.$ (3)

where ***s*** and ***t*** are, respectively, a source and a target for each node, ***s, t ∈ V***. Since we did not separate the seed node sets as a target and source, we assigned each seed node set as both the source and the target sets so that PL can consider all shortest paths between seed nodes. The cost of a path is the sum of the costs of the edges in the path.

## DOMINO

DOMINO utilizes the annotations in Gene Ontology to identify significantly specific communities in the network [[17]](https://paperpile.com/c/GQknHk/xbir). Initially, the Louvain algorithm divides the network into small subnetworks named a slice [[18]](https://paperpile.com/c/GQknHk/IVz4). Overrepresentation analysis detects relevant slices, including initial nodes. During testing relevant slices, DOMINO satisfies one of the criteria described in formulas 4 and 5:

$\frac{k}{m} \geq0 .1$ (4)

$\frac{k}{l} \geq\alpha$ (5)

where ***k*** is the number of initial nodes participating in a given slice, ***m*** is the number of initial nodes in a given reference network, and ***l*** is the number of nodes in the slice.

The single component in each relative slice is handled by solving the Prize Collecting Steiner Tree (PCST) problem, also known as the sub-slice. Sub-slices with more than 10 nodes are partitioned by the Newman–Girvan algorithm into putative modules. The putative modules are individually tested with the hypergeometric test and corrected using the Bonferroni correction. The union of significant and corrected putative active modules constructs the context-specific network or pathway.

## Data preparation

Each pathway within NetPath was independently shuffled, and its nodes were subsequently divided into two equal halves. This procedure was repeated five times. One part of these splits constructs seed nodes, while the other part builds a target node list to be predicted in the reconstructed pathways. All interactions in the pathways, given in NetPath, form the target interactions to be predicted. We recruited HIPPIE v2.2 as a reference network for pathway reconstruction.

The driver genes were randomly divided for each cancer type into five equal portions. Each portion was removed from the most frequently mutated genes. Then, we utilized the remaining frequently mutated genes as a seed node set and HIPPIE v2.2 as a reference so as to infer cancer-specific networks.

## Assessment metrics

We conducted a comparison between GGN and reference networks using topological characteristics and network statistics, including the number of nodes and edges, the highly connected nodes with degrees within the top 20% of all nodes in the reference network, and diameters.

We separately calculated precision, recall, F1 scores, and area under the precision-recall curve (AUPRC) [[19]](https://paperpile.com/c/GQknHk/3vyT) for each pathway. After randomly splitting into two equal pieces five times, we appointed one component as seed nodes and the other as our target nodes. All edges in a given pathway are considered targets in the reference network since we do not provide edge information of pathways to pyPARAGON. Untargeted nodes and edges in the reference network constitute negative nodes and edges.

We also evaluated the performance of the selected tools for the prediction of driver genes from cancer-type-specific networks. We used the most frequently mutated genes, obtained from TCGA and GENIE, as initial nodes. We randomly divided the driver genes retrieved from IntOGen into five equal pieces for each cancer. Since the driver genes are prone to being frequently mutated, we separately removed each partition from the initial nodes. Then, we identified the removed partition of driver genes as positive nodes in cancer-type-specific networks while labeling the remaining nodes from seed and positive nodes in HIPPIE v2.2 as negative nodes. Nonetheless, the lack of ground truth and discrepancies in the cancer-type-specific networks constrain our assessment. The associated interactions of driver genes are inherently unique in patients. In networks, driver-associated nodes and their precise placement are still being unknown, which causes reduced performance metrics. We just focused exclusively on the driver genes in the cancer-type-specific networks by calculating recall and precision scores, and network sizes. We demonstrated precision and recall by selecting the highest F1 scores.

We followed the performance assessment workflow described in [[19]](https://paperpile.com/c/GQknHk/3vyT) while evaluating the performance of the selected tools. In reconstructed pathways or inferred cancer-type-specific networks, target nodes or edges that correctly appear are classified as true positives. Those that incorrectly appear in the inferred networks are labeled false positives. Target nodes or edges that should be present in the reference network but are missing in the inferred networks are considered false negatives, while correctly absent ones in the inferred networks are labeled as true negatives. We employed a grid search strategy to fine-tune the parameters of network reconstruction tools to calculate the Area Under the Precision-Recall Curve (AUPRC). By comparing the optimal precision, recall, and F1 scores derived from various parameter sets, we identified the best configuration for pyPARAGON*.* In OI2, parameter sets were ranged as follows: dummy edge weight *(****ω****),* edge reliability *(****β****)* between 0 and 5 with 0.5 increments, and degree penalty *(****ɣ)*** between 0 and 10 with increment 1. Parameter ***α*** in DOMINO was given between 0 and 1 with 0.05 increments. Similarly, we measured the performance of PL by altering ***k***, the number of shortest paths, between 50-1000 with increments of 50, while the performance of pyPARAGON by ranging the damping factor (**λ**) and flux threshold (**τ**) between 0.05 and 1 with 0.05 increments.

Before the network inference, pyPARAGON first trims the reference network by eliminating redundant interactions with the help of GGN construction. To evaluate the performance of GGN, we calculated the average reduction ratio (RR) of highly connected proteins between the given reference network and GGN. Independent of seed nodes or GGN, we defined the highly connected proteins ***H_R_*** with degrees within the top 20% of all nodes in the reference network [[20]](https://paperpile.com/c/GQknHk/8Z5c): ***h_1_, h_2_, ..., h_n_*** ***∈ H_R_*** for a reference network, the highly connected proteins, ***h_1_, h_2_, ..., h_m_*** ***∈ H_G_*** in GNN, the highly connected proteins, (***h_1_, h_2_, ..., h_m_***) ***∈ H_I_*** in the final inferred pathway, and the highly connected proteins ***h_1_, h_2_, ..., h_p_*** ***∈ H_P_*** in the given pathway, ***H_P_* | *H_I_*** ⊆ ***H_G_*** ⊆ ***H_R_*** ⊆ ***V_R_***. The reduction ratio**s** of the remaining highly connected proteins in GGN, (***H_R_***) and **of** target highly connected proteins in the pathways were separately calculated using the formula 6:

$RR= \frac{{log}_{10}\sum_{i=1}^{m} \frac{{deg}_{R}(h_{i})}{{deg}_{G}(h_{i})}}{m}$ (6)

where ***deg(h)*** is the number of interactions of ***h,*** and ***m*** is the number of highly connected nodes in GGN. We separately calculated the reduction ratio of highly connected proteins for each signaling pathway. Similarly, we calculated the ***RR*** of the highly connected proteins (***H_P_***) in the pathways.

Scale-free networks are widely studied and used in biological networks [[21–23]](https://paperpile.com/c/GQknHk/UBQd+fDm2+k0Sc). Typically, a network is considered scale-free if the percentage of nodes with degree ***k*** follows a power-law distribution, defined in formula 7.

$P_{k} \sim k^{-\gamma}$ (7)

log $P_{k} \sim-\gamma log k$ (8)

where ***k*** is the number of degrees, ***P_k_*** is the probability of ***k***, and ***γ*** is the degree exponent. Some studies on scale-free hypotheses have strong conditions, such as ***2<γ<3*** for the degree distribution of nodes. Thus, we simplified formula 7 with formula 8 to calculate throughout degree distribution on a log scale.

**Tumor-Specific Network Construction**

pyPARAGON constructed the tumor-specific networks for 105 BRCA patients by recruiting differential phosphoproteins as seed nodes**,** and HIPPIE v2.3 as a reference network [[6]](https://paperpile.com/c/GQknHk/RN3o). pyPARAGON uses three parameters: i. significant graphlet types or user-defined graphlets for GGN; ii. the damping factor (***λ***) for the Personalized PageRank (PPR) algorithm; and iii. the scaling factor (***τ***) for the percentage of total flux in GGN. We used the default parameters for tumor-specific networks that maximize the F1 score in performance assessments on the benchmark pathway dataset (NetPath). By default, we used graphlets **G_2_, *G_5_, G_6_, G_7_****,* and ***G_8_*** in GGN construction and set both ***λ*** and ***τ*** to 0.8.

We ran the Louvain method, a fast and heuristic method composed of two iterative steps. (1) Assigning each node to its community, and (2) Interchanging neighbor nodes to find the maximum modularity until no positive gain is achieved [[18]](https://paperpile.com/c/GQknHk/IVz4).

We investigated communities with the over-represented biological processes and KEGG pathways in the inferred networks, which are defined as modules. In this way, we labelled topologically idetified modules with biological annotations. Here, we utilized the hypergeometric distribution, which describes the probabilities of modules associated with the target pool, such as pathways or biological processes. We calculated the p-value using formula 9 [[24]](https://paperpile.com/c/GQknHk/oBzq).

$p= 1-\sum_{i=0}^{k-1} \frac{\left( \frac{M}{i} \right)\left( \frac{M-N}{n-i} \right)}{\left( \frac{N}{n} \right)}$(9)

We define ***M*** as the population size, the number of genes in the reference network; ***N*** as the number of genes in the target pool; ***n*** as the number of genes in the module; and ***k*** as the number of successfully identified genes in the target process. Since multiple modules pointed out the same process, we only selected the most significant module for each biological process or KEGG pathway. A module can be associated with multiple biological processes, or vice versa. Then, we eliminated insignificant communities and their associated biological processes and pathways.

Before clustering patients, we first translated each patient-specific network into a vector space where significant modules with annotations are assigned “1” for a given annotation with a functional module and “0” for unrepresented annotations. Then, these vectors were used for clustering patients and similarity calculations across the tumors. The t-distributed stochastic neighbor embedding (t-SNE) algorithm was used to represent tumor similarities based on two components. We applied t-SNE algorithm to transform the vectors of tumors into two-dimensional data, component-1 and component-2 [[25]](https://paperpile.com/c/GQknHk/f2Ba). The patient groups were determined with agglomerative clustering through the Euclidean distance. Furthermore, we computed the similarity matrix by evaluating the pairwise cosine similarities between the enriched biological processes of each pair of patients by applying the formula 10:

${Sim}_{Cos} = \frac{A x B}{||A|| ||B||}$ (10)

where A and B are vectors of paired patients. Then, we constructed the patient-patient similarity network by adding an edge between patients with similarity scores greater than 0.5.

We used "survminer", an R library, for the Kaplan-Meier analysis considering the overall survival of patient clusters, indicating the percentage of alive patients in the group over time [[26,27]](https://paperpile.com/c/GQknHk/tAk3+9UNJ). We applied the log-rank test, hypothesizing that there are no differences between the survival probabilities of clusters as a null hypothesis. We identified context-specific drugs by mapping targets of therapeutic drugs obtained from the therapeutic target database [[12]](https://paperpile.com/c/GQknHk/NGhk) into patient-specific networks. Additionally, to identify potential drug targets, we extracted TFs in tumor-specific networks regulating cyclin-dependent kinases, mapping them into TRRUST.v2 [[13]](https://paperpile.com/c/GQknHk/Y0lL). Then, we identified FDA-approved therapeutic drugs targeting these TFs.

# References

[1. Alanis-Lobato G, Andrade-Navarro MA, Schaefer MH. HIPPIE v2.0: enhancing meaningfulness and reliability of protein-protein interaction networks. Nucleic Acids Res. 2017; 45:](http://paperpile.com/b/GQknHk/x27S)

[2. Kamburov A, Herwig R. ConsensusPathDB 2022: molecular interactions update as a resource for network biology. Nucleic Acids Res. 2022; 50:D587–D595](http://paperpile.com/b/GQknHk/ZJxt)

[3. Kandasamy K, Sujatha Mohan S, Raju R, et al. NetPath: A public resource of curated signal transduction pathways. Genome Biol. 2010; 11:](http://paperpile.com/b/GQknHk/umH3)

[4. Ellrott K, Bailey MH, Saksena G, et al. Scalable open science approach for mutation calling of tumor exomes using multiple genomic pipelines. Cell Syst. 2018; 6:271–281.e7](http://paperpile.com/b/GQknHk/B6Pb)

[5. Martamp F, Muiamp F, Deu-Pons J, et al. A compendium of mutational cancer driver genes. Nat. Rev. Cancer](http://paperpile.com/b/GQknHk/77NX)

[6. Mertins P, NCI CPTAC, Mani DR, et al. Proteogenomics connects somatic mutations to signalling in breast cancer. Nature 2016; 534:55–62](http://paperpile.com/b/GQknHk/RN3o)

[7. Scrucca L, Fop M, Murphy TB, et al. Mclust 5: Clustering, classification and density estimation using Gaussian finite mixture models. R J. 2016; 8:289–317](http://paperpile.com/b/GQknHk/LBH1)

[8. . Machine Learning with PyTorch and Scikit-Learn: Develop machine learning and deep learning models with Python.](http://paperpile.com/b/GQknHk/uL30)

[9. Kanehisa M, Furumichi M, Sato Y, et al. KEGG for taxonomy-based analysis of pathways and genomes. Nucleic Acids Res. 2023; 51:D587–D592](http://paperpile.com/b/GQknHk/XHCZ)

[10. The Gene Ontology Consortium, Aleksander SA, Balhoff J, et al. The Gene Ontology knowledgebase in 2023. Genetics 2023; 224:](http://paperpile.com/b/GQknHk/RCrm)

[11. Chapple CE, Robisson B, Spinelli L, et al. Extreme multifunctional proteins identified from a human protein interaction network. Nat. Commun. 2015; 6:7412](http://paperpile.com/b/GQknHk/PAbX)

[12. Zhou Y, Zhang Y, Zhao D, et al. TTD: Therapeutic Target Database describing target druggability information. Nucleic Acids Res. 2023;](http://paperpile.com/b/GQknHk/NGhk)

[13. Han H, Cho J-W, Lee S, et al. TRRUST v2: an expanded reference database of human and mouse transcriptional regulatory interactions. Nucleic Acids Res. 2018; 46:D380–D386](http://paperpile.com/b/GQknHk/Y0lL)

[14. Tuncbag N, Braunstein A, Pagnani A, et al. Simultaneous reconstruction of multiple signaling pathways via the prize-collecting steiner forest problem. Journal of Computational Biology 2013; 20:124–136](http://paperpile.com/b/GQknHk/Cfo7)

[15. Ritz A, Poirel CL, Tegge AN, et al. Pathways on demand: Automated reconstruction of human signaling networks. npj Systems Biology and Applications 2016; 2:1–9](http://paperpile.com/b/GQknHk/SnyM)

[16. Gil DP, Law JN, Murali TM. The PathLinker app: Connect the dots in protein interaction networks. F1000Res. 2017; 6:58](http://paperpile.com/b/GQknHk/wveS)

[17. Levi H, Elkon R, Shamir R. DOMINO: a network-based active module identification algorithm with reduced rate of false calls. Mol. Syst. Biol. 2021; 17:e9593](http://paperpile.com/b/GQknHk/xbir)

[18. Blondel VD, Guillaume J-L, Lambiotte R, et al. Fast unfolding of communities in large networks. J. Stat. Mech. 2008; 2008:P10008](http://paperpile.com/b/GQknHk/IVz4)

[19. Arici MK, Tuncbag N. Performance assessment of the network reconstruction approaches on various interactomes. Front. Mol. Biosci. 2021; 8:666705](http://paperpile.com/b/GQknHk/3vyT)

[20. Jin G, Zhang S, Zhang X-S, et al. Hubs with network motifs organize modularity dynamically in the protein-protein interaction network of yeast. PLoS One 2007; 2:e1207](http://paperpile.com/b/GQknHk/8Z5c)

[21. Chen H-H, Hsueh C-W, Lee C-H, et al. SWEET: a single-sample network inference method for deciphering individual features in disease. Brief. Bioinform. 2023; 24:](http://paperpile.com/b/GQknHk/UBQd)

[22. Barabási A-L, Oltvai ZN. Network biology: understanding the cell’s functional organization. Nat. Rev. Genet. 2004; 5:101–113](http://paperpile.com/b/GQknHk/fDm2)

[23. Zhang B, Horvath S. A general framework for weighted gene co-expression network analysis. Stat. Appl. Genet. Mol. Biol. 2005; 4:](http://paperpile.com/b/GQknHk/k0Sc)

[24. Boyle EI, Weng S, Gollub J, et al. GO::TermFinder--open source software for accessing Gene Ontology information and finding significantly enriched Gene Ontology terms associated with a list of genes. Bioinformatics 2004; 20:3710–3715](http://paperpile.com/b/GQknHk/oBzq)

[25. Cieslak MC, Castelfranco AM, Roncalli V, et al. t-Distributed Stochastic Neighbor Embedding (t-SNE): A tool for eco-physiological transcriptomic analysis. Mar. Genomics 2020; 51:100723](http://paperpile.com/b/GQknHk/f2Ba)

[26. Dudley WN, Wickham R, Coombs N. An introduction to survival statistics: Kaplan-Meier analysis. J. Adv. Pract. Oncol. 2016; 7:91–100](http://paperpile.com/b/GQknHk/tAk3)

[27. Scrucca L, Santucci A, Aversa F. Competing risk analysis using R: an easy guide for clinicians. Bone Marrow Transplant. 2007; 40:381–387](http://paperpile.com/b/GQknHk/9UNJ)
